# Supplementary material for: Dipsticks and point-of-care Microscopy to reduce antibiotic use in women with an uncomplicated Urinary Tract Infection (MicUTI): protocol of a randomised controlled pilot trial in primary care
Source: BMJ Open. 2024 Mar 29;14(3):e079345. doi: 10.1136/bmjopen-2023-079345 (PMC10982754; doi:10.1136/bmjopen-2023-079345)
Supplement: Supplementary data [file bmjopen-2023-079345supp001.pdf]

Data management and protection

The recording, disclosure, storage and analysis of personal data within this clinical trial will take place in accordance with the legal provisions of the EU General Data Protection Regulation (GDPR) and of the Bavarian Data Protection Act ('BayDSG') of 15 May 2018. A prerequisite for this is the voluntary agreement of the participants prior to participation.
Data will be recorded and stored as follows.

Data management and protection in each study site

The recruiting academic departments will provide each study site with an investigator site file (ISF), which contains all the documents required for the clinical trial. These include (1) patient questionnaires, (2) eligibility case report forms (CRFs), (3) patient information leaflets with informed consent forms, (4) baseline CRFs, (5) patient diaries, (6) follow-up CRFs, (7) a pseudonymisation list, (8) a patient contact form, and (9) a pre-screening list.
Medical assistants in each study site will be instructed to fill in the pseudonymisation list by consecutively adding the name, birthdays, and date of inclusion of participants. Next to the name, medical assistants will be instructed to write down a patient-ID, containing the letter W or E to identify if the study centre, a three digit number to identify the study site (e.g. 001), and a three digit number to identify the patient (e.g. 001). The pseudonymisation list will be stored separately to all other trial documents and will remain at the study site until the end of data analysis, and destroyed thereafter.
Whenever a new patient is enrolled and once the consent forms are signed:

1) Documents 1, 2, 4-6, and 8 will be provided with the pseudonymized patient-ID.
2) The patient contact information (i. e. telephone number, together with the patient-ID) is written down on the patient contact form and data are entered on REDCap to be transmitted the same day to either the Institute of General Practice of the Friedrich-Alexander Universität Erlangen-Nürnberg (FAU; practices located in or near Erlangen), or to the Institute of General Practice and Family Medicine of the Ludwig-Maximilians University Hospital Munich (LMU; practices located in or near Wuerzburg). It is then stored in the Investigator site file (ISF).
3) The dip-slide is provided with the pseudonymized patient-ID and sent to the Institute of Hygiene and Microbiology of the Julius-Maximilians-University of Würzburg (IMH) via traditional post for microbiological analyses. All results will be reported individually to the treating physician, who will store the report in the ISF. A copy of every report is additionally sent to the trial coordinating centre (UKW).

ISF-data transfer to the academic departments

Researchers and research nurses of the trial teams of the UKW (when practices are located in or near Würzburg) and the FAU (when practices are located in or near Erlangen) perform outreach visits to each study site to collect the paper-based documents (except for the pseudonymisation lists) containing patient data.

Data management and protection at the Academic departments

Research nurses at the trial centres enter the following data in a browser-based electronic Research Data Capture system (REDCap), a password-protected online storage system:

- at the UKW and the FAU they enter all paper-based data of the ISF;
- at the UKW they enter the results of the microbiological analyses performed at the IHM

The Institute of Clinical Epidemiology and Biometry is responsible for providing access to and hosting the EDC-System REDCap, including user management and to ensure data security by regular backups of the database in the context of BayFoNet.

Regular plausibility, validity and consistency checks will be carried out on the described electronic CRFs. Once the data collection is completed, all entries have been made, and final plausibility checks have been carried out, the databases will be closed.

Data management and protection of patients who are not included in the trial

To ensure consecutive enrolment and control for recruitment bias, the following management is planned also for data of patients not subsequently included in the trial:

1) In each study site, all potential participants are approached and listed in an anonymous paper-based pre-screening logs.
2) All approached patients will be asked to complete a symptom questionnaire (document 1, paragraph “Data management and protection in each study site”). Questionnaires filled by included women will be provided with the participant-ID, whereas questionnaires of non-participants will not contain information which could lead to an identification (=*anonymous* data).
3) During each consultation with potentially eligible patients, the GP fills the eligibility CRF (document 2, paragraph “Data management and protection in each study site”). Only data of included women will be provided with the participant-ID. Eligibility CRFs of non-participants will not contain information which could lead to an identification (=*anonymous* data).

These documents will be handed out to the trial teams of the UKW or the FAU together with the ISFs during the final outreach visit.

Storage of paper-based documents at the trial centres

All paper-based documents will be securely stored at the UKW and the FAU and will be accessible only to authorized individuals for ten years and destroyed thereafter.

Storage of human biosamples

No biosamples will be stored in this trial. In the study sites, urine specimens are securely disposed, as in usual clinical practice, immediately after POCTs have been performed. Dip-slides provided with the pseudonymized patient-ID sent to the IMH can be used only for the microbiological analyses as outlined above, and will be disposed thereafter.
