# Supplementary material for: Dipsticks and point-of-care Microscopy to reduce antibiotic use in women with an uncomplicated Urinary Tract Infection (MicUTI): protocol of a randomised controlled pilot trial in primary care
Source: BMJ Open. 2024 Mar 29;14(3):e079345. doi: 10.1136/bmjopen-2023-079345 (PMC10982754; doi:10.1136/bmjopen-2023-079345)
Supplement: Author's manuscript [file bmjopen-2023-079345.draft_revisions.pdf]

BMJ Open is committed to open peer review. As part of this commitment we make the peer review history of every article we publish publicly available.

When an article is published we post the peer reviewers' comments and the authors' responses online. We also post the versions of the paper that were used during peer review. These are the versions that the peer review comments apply to.

The versions of the paper that follow are the versions that were submitted during the peer review process. They are not the versions of record or the final published versions. They should not be cited or distributed as the published version of this manuscript.

BMJ Open is an open access journal and the full, final, typeset and author-corrected version of record of the manuscript is available on our site with no access controls, subscription charges or pay-per-view fees (http://bmjopen.bmj.com).

If you have any questions on BMJ Open's open peer review process please email info.bmjopen@bmj.com

BMJ Open

Dipsticks and point-of-care microscopy to reduce antibiotic use in women with an uncomplicated Urinary Tract Infection (MicUTI): Protocol for a randomised controlled pilot trial in primary care

Journal:	*BMJ Open*
Manuscript ID	bmjopen-2023-079345
Article Type:	Protocol
Date Submitted by the Author:	29-Aug-2023

Complete List of Authors:	Kurotschka, Peter Konstantin; University Hospital Würzburg, Department of General Practice<br>Borgulya, Gábor; University Hospital Würzburg, Department of General Practice<br>Bucher, Eva; University Hospital Würzburg, Department of General Practice<br>Endrich, Isabell; University Hospital Würzburg, Department of General Practice<br>Figueiras, Adolfo; University of Santiago de Compostela, Department of Preventive Medicine and Public Health<br>Gensichen, Jochen; Ludwig Maximilians University Munich, Institute of General Practice and Family Medicine, University Hospital<br>Hay, Alastair; University of Bristol Medical School, Centre for Academic Primary Care<br>Hapfelmeier, Alexander; Technical University of Munich School of Medicine, Department of General Practice and Health Services Research; Technical University of Munich School of Medicine<br>Kretzschmann, Christian; University Hospital Würzburg, Department of General Practice<br>Kurzai, Oliver; University of Würzburg, Institute for Hygiene and Microbiology<br>Lam, Thien-Tri; University of Würzburg, Institute of Hygiene and Microbiology<br>Massidda, Orietta; University of Trento, Department of Cellular, Computational and Integrative Biology<br>Sanftenberg, Linda; University Hospital, Ludwig-Maximilians-Universität, Institute of General Practice and Family Medicine<br>Schmiemann, Guido; University of Bremen, Institute of Public Health and Nursing Research (IPP)<br>Schneider, Antonius; Technical University Munich, Institute of General Practice and Health Services Research<br>Simmenroth, Anne; Julius-Maximilians-Universitat Wurzburg, Department of General Practice<br>Stark, Stefanie; Friedrich-Alexander University Erlangen-Nuremberg, Institute for General Practice and Family Medicine<br>Warkentin, Lisette; Universitätsklinikum Erlangen, Instituteof of General Practice and Family Medicine<br>Ebell, Mark; University of Georgia, Epidemiology and Biostatistics

Gagyor, Ildiko; Julius-Maximilians-Universitat Wurzburg, Department of General Practice

Keywords: Urinary tract infections < UROLOGY, Randomized Controlled Trial, GENERAL MEDICINE (see Internal Medicine)

SCHOLARONE™
Manuscripts

Dipsticks and point-of-care **Mic**roscopy to reduce antibiotic use in women with an uncomplicated Urinary Tract Infection (Mic**UTI**):

Protocol of a randomised controlled pilot trial in primary care

Peter Konstantin Kurotschka[1*], Gabor Borgulya[1], Eva Bucher[1], Isabell Endrich[1], Adolfo Figueiras[2], Jochen Gensichen[3], Alexander Hapfelmeier[4,5], Alastair D Hay[6], Christian Kretzschmann[1], Oliver Kurzai[7], Thien-Tri Lam[7], Orietta Massidda[8], Linda Sanftenberg[3], Guido Schmiemann[9], Antonius Schneider[4], Anne Simmenroth[1], Stefanie Stark[10], Lisette Warkentin[10], Mark H. Ebell[11], Ildikó Gágyor[1] on behalf of the Bavarian Practice-Based Research Network (BayFoNet)

[1] Department of General Practice, University Hospital Würzburg, Würzburg, Germany

[2] Department of Preventive Medicine and Public Health, University of Santiago de Compostela, Santiago de Compostela, Spain

[3] Institute of General Practice and Family Medicine, University Hospital, Ludwig-Maximilians-Universität München, Munich, Germany

[4] Institute of General Practice and Health Services Research, School of Medicine, Technical University of Munich, Munich, Germany

[5] Institute of AI and Informatics in Medicine, School of Medicine, Technical University of Munich, Munich, Germany

[6] Centre for Academic Primary Care, Bristol Medical School: Population Health Sciences, Department of Community Based Medicine, University of Bristol, Bristol, United Kingdom

[7] Institute for Hygiene and Microbiology, University of Würzburg, Würzburg, Germany

[8] Department of Cellular, Computational and Integrative Biology, University of Trento, Trento, Italy

[9] High-Profile Area of Health Sciences, University of Bremen, Bremen, Germany

[10] Institute of General Practice, Friedrich-Alexander-Universität Erlangen-Nürnberg, University Hospital Erlangen, Erlangen, Germany

[11] Department of Epidemiology and Biostatistics, College of Public Health, University of Georgia, Athens, Georgia, United States

*Author to whom the correspondence should be addressed: Kurotschka_p@ukw.de

Word count: 3800

Keywords: Urinary Tract Infections; Women; General Practice; Randomized Controlled Trial; Anti-Infective Agents; Microscopy

For peer review only - http://bmjopen.bmj.com/site/about/guidelines.xhtml

Abstract

Introduction: Uncomplicated urinary tract infections (uUTIs) in women are common infections encountered in primary care. Evidence suggests that rapid point-of-care tests (POCTs) to detect bacteria and erythrocytes in urine at presentation may help primary care clinicians to select women with uUTIs in whom antibiotics can be withheld without affecting clinical outcomes. This pilot study aims to provide preliminary evidence on whether a POCT informed management of uUTI in women can safely reduce antibiotic use.

Methods and analysis: MicUTI (Microscopy in Urinary Tract Infections) is an open-label two-arm parallel cluster-randomised controlled pilot trial. Twenty general practices affiliated with the Bavarian Practice-Based Research Network (BayFoNet) in Germany will be randomly assigned to deliver patient management based on POC-tests or based on usual care. POC-tests used are phase-contrast microscopy to detect bacteria and urinary dipsticks to detect erythrocytes in urines. In both arms, urine samples will be obtained at presentation for POC-tests (intervention arm only) and microbiological analysis. Women will be followed-up for 28 days from enrollment using self-reported symptom diaries, telephone follow-up, and a review of the electronic medical record. Primary outcomes are feasibility of patient enrollment and retention rates per site, which will be summarised by means and SDs, with corresponding confidence and prediction intervals. Secondary outcomes include antibiotic use for UTI at day 28, time to symptom resolution, symptom burden, the number of recurrent and upper UTIs and re-consultations, and diagnostic accuracy of POC-tests vs. urine culture as reference standard. These outcomes will be explored at cluster- and individual-levels using descriptive statistics, two-sample hypothesis tests and mixed effects models or generalized estimation equations.

Ethics and dissemination: The University of Würzburg institutional review board approved MicUTI on December 16, 2022 (protocol n. 109/22-sc). Findings will be disseminated through peer-reviewed publications, conferences, reports addressed to clinicians, and local citizen´s forums.

Trial registration: ClinicalTrials.gov NCT05667207.

Keywords: Urinary Tract Infections; Cystitis; Female; Humans; Anti-Bacterial Agents; Randomized Controlled Trial; Pragmatic Clinical Trial; Point-of-Care Testing; Microscopy; Urinalysis; Bacteriuria; Hematuria

Strenghts and limitations of this study

- This study will inform the design and conduct of confirmatory RCTs to evaluate the effects of a POCT guided management strategy on the use of antibiotics in women with suspected uncomplicated UTI in primary care.
- The open-label cluster-RCT design implies that participants cannot be identified before randomisation, so that post-randomisation recruitment may represent a source of selection bias.
- The execution and interpretation of microscopy test results may differ among physician assistants, affecting diagnostic accuracy measurements and treatment outcomes. This will be counteracted by providing all intervention practices with standardised ad-hoc competency-based trainings.

For peer review only - http://bmjopen.bmj.com/site/about/guidelines.xhtml

Introduction

Uncomplicated urinary tract infections (UTIs) are a common reason for adult women to consult a healthcare professional and a major driver of antibiotic prescriptions in the community.[1] Up to 95% of women presenting to their general practitioner (GP) receive antibiotics, despite uncomplicated UTIs (uUTIs) being self-limiting in up to 50% of cases.[1 2]
As antibiotic prescriptions are closely related to the emergence of resistant microorganisms, reducing unnecessary use of these drugs in uUTI management is important to counteract the rise of antimicrobial resistance (AMR).[3-5]

Randomised controlled trials (RCTs) were conducted to establish whether alternatives to immediate antibiotic treatment, such as delayed prescriptions, herbal formulations, or non-steroidal anti-inflammatory drugs reduce antibiotic use without significant harm.[6-11] All strategies were highly effective in reducing antibiotic use over usual care. The downside was that, if compared to immediate antibiotics, the alternative treatment strategies showed an increased risk of longer symptom duration, higher symptom burden, incomplete recovery, febrile UTI, pyelonephritis and antibiotic use at follow-up.[12]
A recent meta-analysis confirmed these findings analysing individual participant data of 3524 patients from eight primary care RCTs.[13] In addition, the authors of the latter meta-analysis focused on prognostic and moderating factors of treatment effects. They found that, in women treated without antibiotics, incomplete recovery was more likely if, at baseline, both erythrocytes are found in urine and women had a positive urine culture (OR 4.68, Bayesian credible interval [CI] 2.07-10.77). If only the urine culture was positive, incomplete recovery was likely with OR 2.24 (CI 1.00-5.17), while if only the test for erythrocytes was positive, OR was 2.60 (CI 1.08-6. 32). In contrast, the authors found no significant difference in the likelihood of incomplete recovery between non-antibiotic strategies and immediate antibiotics if both tests were negative (OR 0.82, CI 0.33-1.95). Moreover, positive urine culture results and erythrocytes in urine were found to be independent predictors of complications such as pyelonephritis, febrile UTI, and of antibiotic use at follow-up.[13]

These findings show a clear benefit of immediate antibiotics in all cases in which the woman´s urine culture is positive and erythrocytes are found in urine. However, whereas simple and affordable tests exist to detect erythrocytes at the point-of-care (POC) rapidly, namely urinary dipsticks [14], standard urine cultures are not useful for immediate clinical decisions, as they require 48 hours or more for providing definitive results.[15-17] This led to studies that evaluated portable devices to perform urine culture, which could deliver quicker results than standard cultures and possibly lead to a more accurate diagnosis than the usual diagnostic approach based only on symptoms and dipstick test results. The multinational POETIC RCT found that POC urine culture and susceptibility testing was not effective at reducing immediate antibiotic prescriptions.[18] A Danish RCT found that adding POC susceptibility testing to POC-culture is unlikely to improve the appropriateness of treatment decisions in women with uUTI.[19] One explanation for these poor results may be, that POC-cultures need 24 hours to deliver results and, given the prognostic and moderating effects of a positive urine culture, i.e. significant

For peer review only - http://bmjopen.bmj.com/site/about/guidelines.xhtml

bacteriuria, the treatment decision is best to be made immediately during index consultation.[13]

Thus far, urine microscopy is the only rapid POC-test (POCT) able to directly detect bacteria in urine that was evaluated in a general practice setting. Among the various microscopic methods, phase-contrast microscopy does not require centrifugation or other time-consuming specimen preparation, thus being easier to implement in practice than other microscopic methods.[20] Several studies have investigated its diagnostic accuracy in detecting UTI in adult outpatients showing promising, although conflicting results. Reported sensitivity varied between 74% and 95%, while specificity ranged between 63% and 97%.[21-24] Nevertheless, although a recent systematic review concluded that microscopy "seems to be an accurate, valid and feasible screening-test for bacteriuria in patients with symptoms of UTI in general practice", it acknowledged that there is still a lack of solid evidence to determine its accuracy to diagnose UTIs.[25]
Diagnostic RCTs have the advantage over traditional diagnostic studies of providing information on test accuracy but also on whether the expected differences in accuracy over usual care are clinically relevant.[26] To date, the effect of a diagnostic and treatment strategy guided by POCTs for bacteria and erythrocytes in urine on antibiotic use in women with suspected uUTI has not been studied. This pilot RCT aims to inform about the feasibility and sample size of a full-scaled diagnostic RCT in general practice and to provide initial data on the effect of the POCT guided diagnostic and treatment algorithm on antibiotic use. POC-tests used are phase-contrast microscopy to detect bacteria and urinary dipsticks to detect erythrocytes in urines.

Methods and analysis

Design

MicUTI (Microscopy in Urinary Tract Infections) is a pragmatic open-label, two-arm parallel cluster randomised pilot trial. General practices will be randomly allocated to either the POC-test based management strategy (intervention) or standard care management (usual care). For the trial duration (6 months), practices will be asked to systematically recruit women aged 18 to 70 years with symptoms suggesting a uUTI (dysuria, frequency/urgency of micturition, nicturia, with or without lower abdominal pain) to be included in the trial.

Objectives

Primary objective

The primary objective is to assess the feasibility of a full-scaled diagnostic RCT. Primary endpoints are patient recruitment efficacy and percentage of retention in the trial. Recruitment efficacy is calculated as the number of participants enrolled per site over six months of trial duration, and retention as the percentage of complete follow-ups per site over 28 days.

For peer review only - http://bmjopen.bmj.com/site/about/guidelines.xhtml

Secondary Objectives

The secondary objectives of MicUTI are to compare the intervention and control practices with regard to the following endpoints

1. Total antibiotic use: number of antibiotic prescriptions per patient with UTI within 28 days
2. Defined daily doses of the prescribed antibiotics per patient with UTI within 28 days
3. Inappropriate antibiotic use: the percentage of patients with symptoms of UTI who were prescribed antibiotics among those with negative urine cultures
4. Number of immediate and delayed antibiotic prescriptions for uUTI at initial consultation
5. Number of early relapses of UTI (days 0-14)
6. Number of recurrent UTIs (days 15-28)
7. Number of upper UTIs within 28 days
8. Number of consultations due to UTI (or symptoms of UTI) within 28 days
9. Time to symptom resolution defined as a maximum of 1 point in each of the UTI-SIQ-8 items[34]
10. Total symptom burden on days 0-7 (area under the curve of the UTI-SIQ-8 total symptom score)
11. Diagnostic accuracy of microscopy +/- dipstick compared to the standard (urine culture)

A further objective of this study is to evaluate the feasibility and acceptability of POC-microscopy to detect bacteria in urine and the training sessions addressed to medical assistants in the intervention arm practices.

Setting

MicUTI will be conducted in a primary care research network based in the German Federal State of Bavaria, the Bavarian Practice Based Research Network (BayFoNet). Three out of the five university departments of general practice part of the BayFoNet are involved in MicUTI. These are the Department of General Practice of the University Hospital Würzburg (hereafter abbreviated asUKW), which is the trial coordinating center, the Institute of General Practice of the Friedrich-Alexander Universität Erlangen-Nürnberg (hereafter abbreviated as FAU) and the Institute of General Practice and Family Medicine of the Ludwig-Maximilians University Hospital Munich (hereafter abbreviated as LMU), who collaborate in the recruitment of study sites and data collection. An accompanying mixed-methods process evaluation will assess the implementation of this trial into the BayFoNet and further investigate its feasibility and acceptability to general practice teams. Details of the process evaluation study are published elsewhere.[27]

Recruitment of study sites

The UKW and the FAU will recruit 20 general practices located in the Federal State of Bavaria (Würzburg and Erlangen and surrounding areas) to participate in the trial through postal

For peer review only - http://bmjopen.bmj.com/site/about/guidelines.xhtml

invitations, institutional advertisements such as newsletters and website announcements, and public conferences with GP practice teams.

Randomisation of study sites

General practices participating in the trial (hereafter: study sites) will be asked to provide the number of consulting patients over the last year to determine practice size. Study sites of larger size are foreseeably able to recruit more patients for the duration of the trial, so that practice size will be balanced between the two arms (each with ten practices) using minimisation.[28] The minimisation algorithm is centrally programmed in Python by the study statistician. To mask the trial statistician (GB) to intervention / control assignments, the minimisation algorithm will be executed by the trial´s statistical consultant based at the Technical University of Munich (AH), who is not involved in the conduction of the trial. Intervention / control assignments of practices will be communicated to the UKW and the FAU, who then communicate to the study sites their allocation. To minimise selection bias, study sites were identified for inclusion in the trial prior to randomisation.

Patient recruitment/enrollment

Identification of women eligible for inclusion

In all study sites, at presentation, medical assistants will consecutively approach all adult women presenting with two or more symptoms suggestive for an acute uUTI (dysuria, frequency, urgency, nocturia, lower abdominal pain) to identify trial participants. To ensure consecutive enrollment and control for selection bias, all potential participants will be approached and listed in paper-based patient pre-screening logs at each study site. Interested women will be asked to complete the validated symptom questionnaire UTI-SIQ-8 [29] and will be provided with a comprehensive patient information leaflet which details trial procedures in lay language.

The GP will exclude from participation all women with one or more of the following:

- Signs of a complicated UTI (anamnesis of fever, chills or flank pain)
- Clinically relevant immunosuppression (i.e., current use of any immunosuppressive therapy, congenital or acquired disorders of immunity)
- Acute or chronic functional or anatomical variations in the urinary tract except for chronic kidney failure with eGFR > 45 ml/min
- Permanent bladder catheter or use of bladder catheter within the past two weeks
- UTI within the past two weeks
- Use of any antibiotic within the past two weeks
- Accommodation in a nursing home or hospital stay within the past two weeks
- Severe neurologic, psychiatric illness, severe dementia or severe substance use disorder
- Other severe diseases
- Being unable to understand the informed consent or to complete the patient diary
- Known pregnancy

For peer review only - http://bmjopen.bmj.com/site/about/guidelines.xhtml

Women who consent will be asked to sign an informed consent form, which also contains data protection regulations.

Intervention arm

GPs whose practice is allocated to the intervention will have their management guided by POCTs, namely phase-contrast microscopy and urinary dipsticks, for all patients consenting to participation.

Point-of-care tests

Medical assistants will perform microscopy using phase-contrast microscopes ("Primostar", Carl Zeiss Suzhou Co.®, Suzhou, China) to examine 7 µl clean-catch mid-stream urine (MSU) without prior centrifugation at 400x magnification in a precision counting chamber (Fast-Read® 102 slides, Biosigma s.r.l., Cona (VE), Italy) to detect bacteria. A test is deemed positive if more than a few bacteria of the same shape (rod or cocci) or if many bacteria of different shapes (rod and cocci) are detected per high power field.

Urinary dipstick analysis will be performed using COMBUR5-Test® (Roche Diagnostics GmBH, Mannheim, Germany) to examine 50 ml MSU to detect erythrocytes. According to the manufacturer instructions, the COMBUR5®-test strip will be dipped in the urine sample for about one second, wiped against the rim of the vessel to remove excess urine and read manually after 60 seconds by comparing the colour of the detection pad of the strip with the colour scale on the test strip vial. A positive test result is defined as the color change of the strip corresponding to 1+ erythrocytes or greater.

All the analyses will be carried out immediately after the MSU specimen collection.

Training of study sites in trial procedures

Medical assistants are provided with ad-hoc training to approach potentially eligible women consecutively, and to perform POCTs according to the study protocol by dedicated staff. It will be delivered face-to-face prior to patient recruitment in a three-hour session (baseline session), as well as three months after the start of patient recruitment (refresher session), following the principles of competency-based medical education.[30] The baseline training session entails the following: (1) Guideline knowledge on UTI in women to allow medical assistants to identify potentially eligible women in each study site consecutively; (2) POCT training to perform and interpret the results of phase-contrast microscopy and urinary dipsticks according to the study protocol. The knowledge and skills gained and retention, the feasibility, as well as the acceptability of the training sessions and the use of phase-contrast microscopes in daily practice, will be assessed through a questionnaire addressed to medical assistants before, immediately after, and three months after the training session, and through face-to-face semi-structured interviews, three months after the training session. Interviews will be conducted following a pre-planned interview-guide informed by the Consolidated Framework for Implementation Research (CFIR).[31] Practical tests at the microscope directly

For peer review only - http://bmjopen.bmj.com/site/about/guidelines.xhtml

after the training session and after three months assess each medical assistant's acquisition and retention of skills.

Point-of-care test guided treatment algorithm

Based on the prognostic and moderating effects on treatment outcomes of erythrocytes and bacteria in urine [13], GPs will be encouraged to apply the following treatment algorithm (figure 1) to consenting women, taking their preferences into account:

1. If POCTs are positive for bacteria by microscopy and/or for erythrocytes by dipsticks, the GP issues, at their own clinical judgement, a delayed or immediate prescription for an antibiotic. In the MicUTI intervention, a delayed prescription can be issued by GPs, at their discretion, in either way: the prescription is handed out to the patient with the advice to redeem it only when symptoms do not improve or worsen in 48 hours, or it is withheld in the GP practice. It can be handed out if the patient present again with complaints.
2. If POCTs are negative for bacteria and erythrocytes, the GP advises for self-help remedies according to national guidelines and to do without antibiotics.[32 33]

Figure 1. Treatment algorithm in the intervention arm

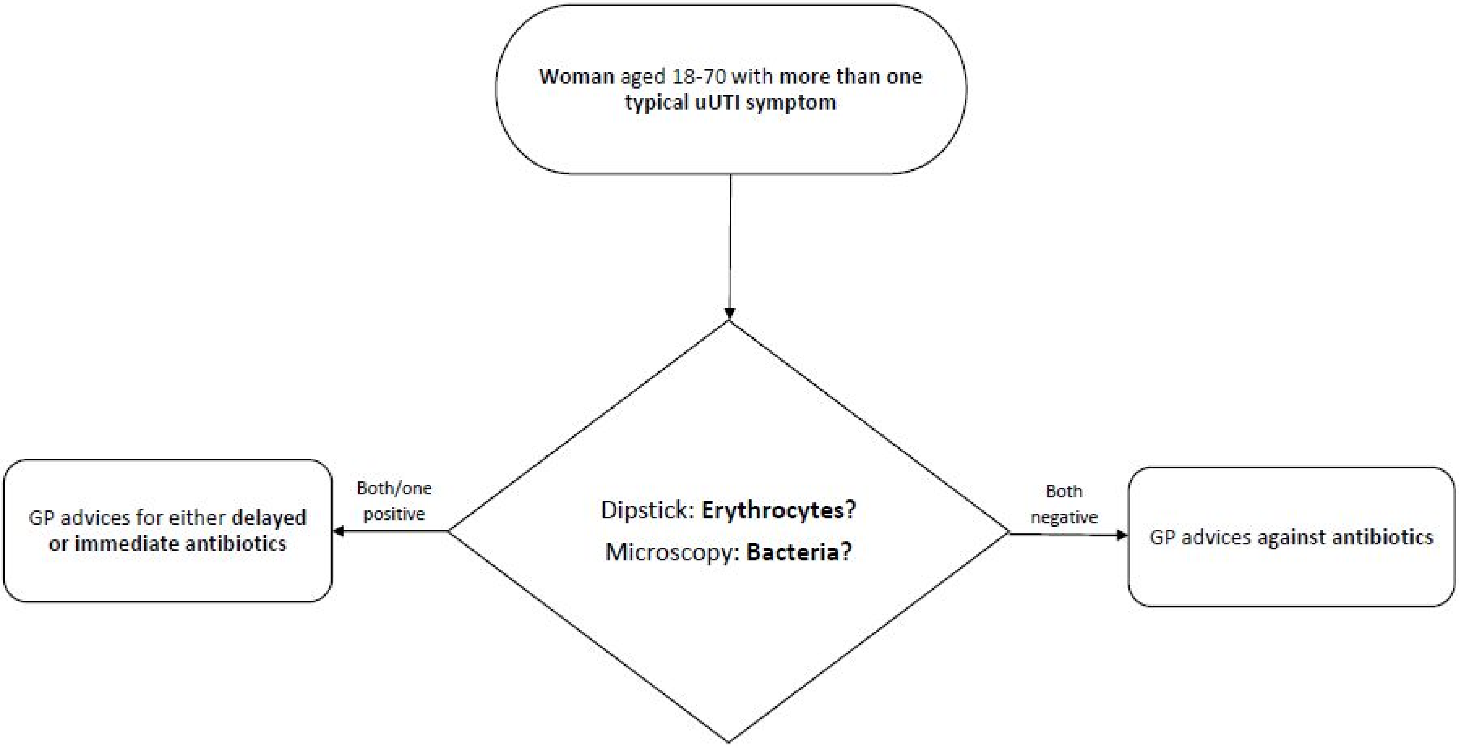

Abbreviations. uUTI = uncomplicated urinary tract infection; GP = general practitioner

Data collection

<u>Baseline</u>

Baseline data will be collected during routine consultation. The medical assistant records microscopy and dipstick test results, clinical features, and the GP´s management decisions,

including antibiotics prescribed on a baseline paper-based CRF. In addition, the medical assistant inoculates a dipslide (see paragraph “microbiological analyses”) to be send via post on the same day to the central research laboratory based at the Institute of Hygiene and Microbiology of the Julius-Maximilians-University of Würzburg (IHM) for microbiological analysis.

Follow-up

Follow-up data will be collected through a self-directed patient diary, follow-up telephone calls at day 28 from inclusion, and follow-up in the GP practice in case of re-consultation. In addition, an electronic medical records (EMR) review will also be performed to double check for missing follow-up data.

Patient diary. Women will be asked to rate their daily symptoms and impairment due to UTI on a scale from 1 (no symptoms/impairment at all) to 5 (very strong symptoms/impairment) and to write down the antibiotics taken in a diary for at least seven days (or up to 14 days if symptoms last longer).[35] Enough space in the diary is left for women to take extra notes. They will be asked to return the completed diary to their GP practice using a pre-paid envelope or in person. Research nurses at the FAU and the LMU will perform telephone calls on day 2-4 from inclusion to remind enrolled women to fill out and return the diary.

Follow-up telephone calls. Calls will be performed by research nurses at the FAU and the LMU on day 28 from inclusion to collect data on symptoms, antibiotic use due to UTI, recurrences, and re-consultations with UTI symptoms.

Follow-up in the GP practice. Every time a woman re-consults for UTI within 28 days, the GP records clinical features, management decisions, and antibiotics prescribed on follow-up paper-based CRFs.

Electronic medical records review. Researchers from the UKW and the FAU will perform outreach visits at the end of the patient recruitment (6 months) in each of the study sites. Paper-based CRFs will be collected, and data will be controlled for completeness. To double-check data entries for accuracy and completeness, each enrolled woman's electronic medical records (EMRs) will be enquired on re-consultations with UTI symptoms, recurrences, antibiotics prescribed due to UTI, and upper UTI until day 28 from inclusion.

Control arm–usual care

Practices in the control arm will not necessarily have their management guided by POCTs. They will perform usual care. The treatment decision is usually based on symptoms with or without dipstick test results (i.e., erythrocytes, leukocytes, nitrites). Procedures are the same as those outlined above, except for POCTS: microscopy only pertains to the intervention arm.

Table 1 and figure 2 summarise trial procedures.

For peer review only - http://bmjopen.bmj.com/site/about/guidelines.xhtml

Table 1. Schedule of MicUTI trial procedures according to the Standard Protocol Items: Recommendations for Interventional Trials (SPIRIT) guideline.[35]

TIMEPOINT (Day 0 = patient enrolment) *Procedures*	< 0	Day 0 Baseline	Day 1-7	Day 28	End of patient enrollment[a]
**ENROLMENT:**					
Practice recruitment	X				
Randomisation	X				
Allocation	X				
Training	X				
Screening for potential inclusion		X			
Formal eligibility assessment		X			
**INTERVENTION:**					
Baseline consultation[1]		X			
POCTs[2]		X			
Urine sent to laboratory[1]		X			
**ASSESSMENTS:**					
Practice size	X				
Paper based-CRF[3]		X			
Patient diary[4]			X		
Follow-up telephone calls				X	
EMR review[5]					X

[a] End of patient enrollment in each practice

[1] Applies to intervention and control arm practices

[2] Applies only to intervention arm practices

[3] In case of re-consultation for UTI on days 1-28, paper-based follow-up CRFs are used

[4] Follow-up on days 1-7 or up to day 14 if symptoms last longer or recur.

[5] Follow-up on days 1-28 (CRF completeness and accuracy check)

For peer review only - http://bmjopen.bmj.com/site/about/guidelines.xhtml

Figure 2. Flow diagram of the MicUTI-trial procedures

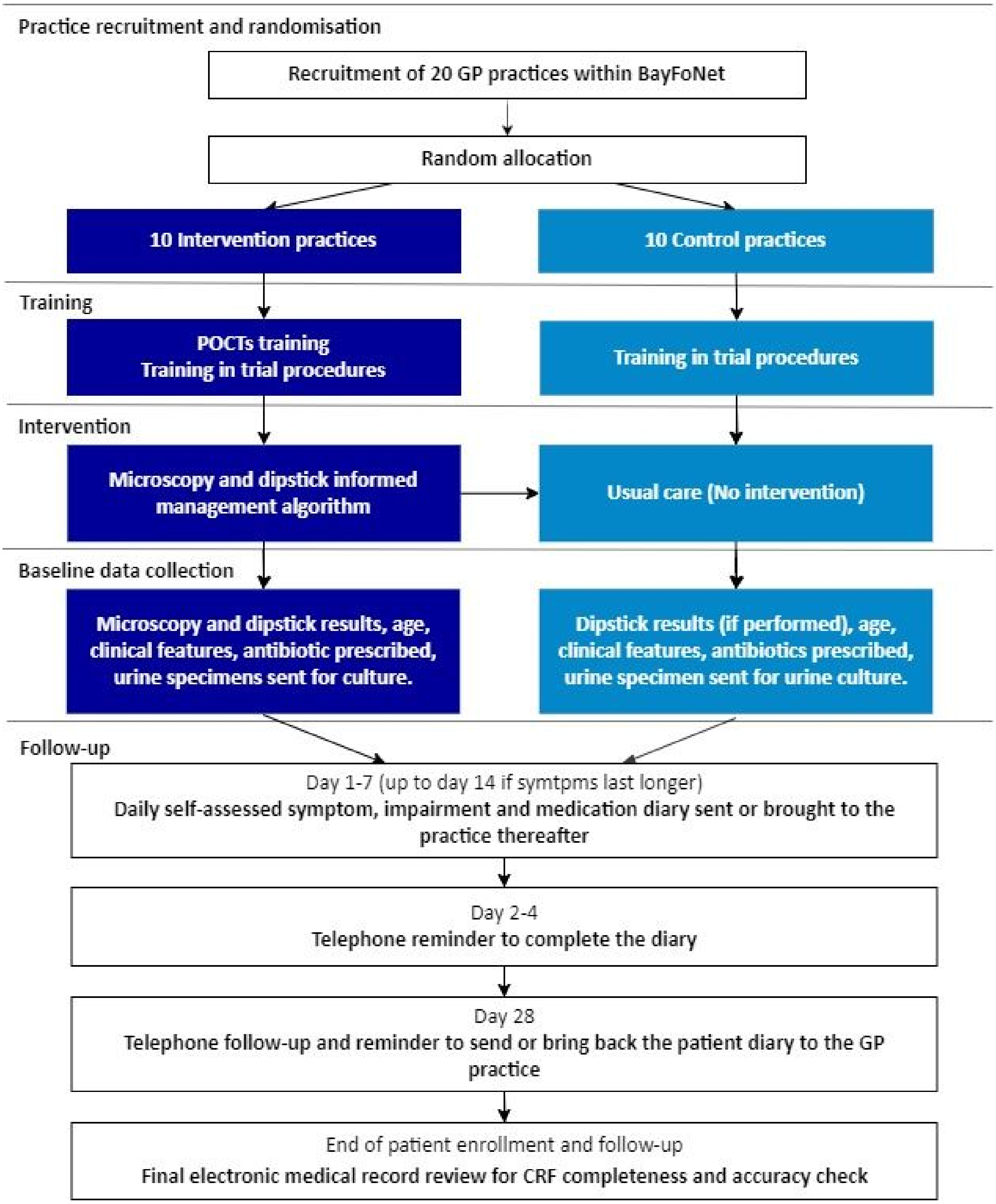

Abbreviations. POCTs = Point-of-Care tests; GP = general practitioner; CRF = case report form

Microbiological analyses

For cultural urine diagnostics, at each study site, medical assistants immerse a dipslide, i. e. a culture medium carrier (Uricult® Plus, Roche Diagnostics GmbH, Mannheim, Germany) into the test urine until the agar surface is completely covered. After removal from the urine, the carrier is put into a sterile transport tube for transportation via post to the IHM. Upon arrival,

For peer review only - http://bmjopen.bmj.com/site/about/guidelines.xhtml

the carrier is incubated at 35 ± 2°C for 16 to 24 h. If bacterial growth is detected, the subsequent analysis is carried out according to the established diagnostic procedures of the accredited medical laboratory. Briefly, the analysis includes determination of the overall bacterial count on the Cystine Lactose Electrolyte Deficient–agar (CLED) plates, and those of Gram-negative bacteria (on MacConkey agar plates) and of *Enterococcus spp.* (on the *Enterococcus* agar plates). Bacteria are subcultured on Columbia Sheep Blood agar plates and MacConkey agar plates to be subsequently identified by mass spectrometry (VITEK MS, BioMérieux, Marcy-l'Etoile, France). Susceptibility testing of pathogenic bacteria is done using VITEK 2 (bioMérieux, Marcy-l'Etoile, France) and interpreted according EUCAST.
To define a significant growth in the MSU specimens, the criteria specified in the updated 2020 German infectious disease and microbiology laboratory quality standards will be used.[34]

Statistical analyses
Descriptive statistics will be calculated for all variables per site and as summary measures across sites. Continuous and count variables will be summarised by mean and SD, or median and IQR in case of skewed data. Binary and categorical variables will be summarised through counts and percentages. Confidence intervals and prediction intervals will be calculated for the two feasibility endpoints (recruitment efficacy and percentage of retention in the trial) in addition.
We acknowledge that this feasibility RCT is not powered to detect any statistically significant effects. Nevertheless, explorative analyses will be undertaken at the cluster-level and the individual-level to evaluate the effects of the intervention on the secondary outcome variables using two-sample hypothesis tests, random effects models and generalized estimation equations, as appropriate. Alongside point-estimates, p-values and 95% confidence intervals will be reported.

Missing data will be explored to understand why they are unavailable; when missing data are due to incomplete follow-up survival or censored regression analyses will be considered, otherwise multiple imputation with chained equations or Bayesian imputation will be applied. The intraclass correlation coefficient (ICC) will be calculated for the secondary endpoints to inform subsequent sample size and power calculations.

All the analyses will be undertaken using statistical software packages Stan, R, and STATA.

Qualitative data analysis of interviews addressed to medical assistants
All interviews will be de-identified, transcribed verbatim, and analysed by at least two researchers inductively via content-analysis inspired by Kuckartz.[35] The analysts perform first line-by-line coding after extensive reading of the interviews (familiarisation). While iteratively refining the code labels, they are grouped to generate categories and subcategories. In subsequent iterations of the process, categories and subcategories are refined in light of the research question and are summarised in a final report of the analyses.

For peer review only - http://bmjopen.bmj.com/site/about/guidelines.xhtml

Sample size considerations

An ad hoc analysis undertaken on claims data provided by the Bavarian health insurance union (Kassenärztliche Vereinigung) covering all diagnosed UTIs in ambulatory care from 2015 to 2019 showed that each GP had an average of 60 encounters for unUTI in women aged 18-70 per year, with only slight seasonal fluctuations (data not shown). Considering that some of these encounters occur in out-of-hours care and that not all of the patients presenting with unUTI are going to be eligible, we expect that 200 patients in both arms (i. e. 100 patients per arm = an average of 10 enrolled patients in each of the 20 clusters) is a reasonably achievable sample within six months of trial duration.

Assuming that the percentage of complete follow-up is 75% in the population the patients are sampled from, a simulation study showed that the 200 patients of the feasibility trial enable the estimation of the percentage of complete follow-up with a 95% confidence interval of width of less than 13% with a likelihood of 0.90.

Patient and public involvement

A citizens' forum was established within the Bavarian Practice Based Research Network (BayFoNet) framework. Invited are all citizens who are interested in contributing to research projects of the Department of General Practice (University Hospital Würzburg). All important steps that are necessary for different projects and studies are discussed with the participants. MicUTI has been discussed with the citizens twice so far. After the presentation of the study, the participants discussed whether therapy for uUTI without antibiotics would be conceivable. In addition, the members gave feedback on the comprehensibility and clarity of the patient diary and the patient information.

Furthermore, feedback was given in the following meeting of the citizens' forum on how the citizens' comments were incorporated into the further work.

Ethics and dissemination

The study will be conducted in accordance to the Declaration of Helsinki in its current version.[36] The University of Würzburg institutional review board approved MicUTI on December 16, 2022 (protocol n. 109/22-sc). The recording, disclosure, storage and analysis of personal data within this clinical trial will take place by the legal provisions of the EU General Data Protection Regulation (GDPR). To protect confidentiality, all data will be collected and stored psudonymised. Pseudonymisation lists will be stored in a secure place at each study sites separately from CRFs.[37]

A trial steering committee is established. The committee is composed by the principal investigator (IG), the study coordinator (PK) and by internationally renowned experts in the implementation and conduction of pragmatic RCTs in primary care and research methodology (AH, AF, AH, GS, MHE). The committee provides an overall methodological supervision of the study and safeguards the interest of study participants. Any of its members will be informed of amendments to the protocol.

The implementation of MicUTI will generate the needed knowledge to plan a confirmatory, full-scaled trial, intended to reduce the clinical equipoise about rapid POCTs used to inform

For peer review only - http://bmjopen.bmj.com/site/about/guidelines.xhtml

the clinical management of uUTIs in general practice. Study results will be disseminated through peer-reviewed publications, academic conferences, a report addresses to participating GPs and through a summary report in lay language addressed to patients and the public.

Author contributions. PK and IG conceived the study and are primarily responsible for the study design. GB, together with PK, planned the randomisation, the statistical analyses and the sample size. PK wrote the protocol consulting IG, AH, AF, GS, GB, AH, AS, ASc and MHE (trial design), OM, TTL, and OK (point-of-care tests and microbiological analyses), IG and CK (data protection and PPI), EB, IE, StS, LW, LS, and JG (local implementation, data collection). All authors read and approved the final version of the manuscript.

Funding. The present study is entirely funded by the German Federal Ministry of Education and Research (BMBF) grant Nr. 01GK1903A "Development of a sustainable structure for practice-based research networks for the strengthening of general practice".
The funder had no role or authority in the design, or in the writing of the protocol. Moreover, the funder will have no role or authority in data collection, data management, data analysis, interpretation, reporting, writing up or publication of findings.

Data availability. Raw data will be available upon reasonable request from the corresponding author after the final publication of trial results.

Competing interests. None declared.

Acknowledgements. We are grateful for all participating general practices. A particular thank goes to Maike Ermster, Waltraud Flederer, Merle Klanke, Anna-Lena Schnaidt and Petra Hagenbusch for their help in the field implementation. We also thank Alice Serafini, MD, for her valuable advices in the planning of the field implementation.
PK and EB, as PhD and MD candidates respectively, are supported by funds of the Bavarian State Ministry of Science and Arts and the University of Würzburg to the Graduate School of Life Sciences (GSLS), University of Würzburg.
Bavarian Practice-Based Research Network (BayFoNet) members include, in alphabetical order: Andrea Baumgärtel, Marlene Becker, Melanie Bößenecker, Tobias Dreischulte, Stefanie Eck, Isabell Endrich, Ildikó Gágyor, Jan Gehrmann, Jochen Gensichen, Alexander Hapfelmeier, Susann Hueber, Merle Klanke, Christian Kretzschmann, Thomas Kühlein, Peter K. Kurotschka, Klaus Linde, Klara Lorenz-Dant, Selina Nadler, Marco Roos, Linda Sanftenberg, Antonius Schneider, Stefanie Stark, Til Uebel, Fabian Walter.

References

1. Butler CC, Hawking MK, Quigley A, et al. Incidence, severity, help seeking, and management of uncomplicated urinary tract infection: a population-based survey. *Br J Gen Pract* 2015;65(639):e702-7. doi: 10.3399/bjgp15X686965 [published Online First: 2015/09/29]
2. Hoffmann T, Peiris R, Mar CD, et al. Natural history of uncomplicated urinary tract infection without antibiotics: a systematic review. *Br J Gen Pract* 2020;70(699):e714-e22. doi: 10.3399/bjgp20X712781 [published Online First: 2020/09/23]
3. Costelloe C, Metcalfe C, Lovering A, et al. Effect of antibiotic prescribing in primary care on antimicrobial resistance in individual patients: systematic review and meta-analysis. *BMJ* 2010;340:c2096. doi: 10.1136/bmj.c2096 [published Online First: 2010/05/21]
4. Laxminarayan R. The overlooked pandemic of antimicrobial resistance. *The Lancet* 2022;399(10325):606-07. doi: 10.1016/S0140-6736(22)00087-3

For peer review only - http://bmjopen.bmj.com/site/about/guidelines.xhtml

5. Murray CJL, Ikuta KS, Sharara F, et al. Global burden of bacterial antimicrobial resistance in 2019: a systematic analysis. *The Lancet* 2022;399(10325):629-55. doi: 10.1016/S0140-6736(21)02724-0
6. Gágyor I, Bleidorn J, Kochen MM, et al. Ibuprofen versus fosfomycin for uncomplicated urinary tract infection in women: randomised controlled trial. *BMJ (Clinical research ed)* 2015;351:h6544. doi: 10.1136/bmj.h6544
7. Gágyor I, Hummers E, Schmiemann G, et al. Herbal treatment with uva ursi extract versus fosfomycin in women with uncomplicated urinary tract infection in primary care: a randomized controlled trial. *Clin Microbiol Infect* 2021;27(10):1441-47. doi: 10.1016/j.cmi.2021.05.032 [published Online First: 20210607]
8. Little P, Moore MV, Turner S, et al. Effectiveness of five different approaches in management of urinary tract infection: randomised controlled trial. *Bmj* 2010;340:c199. doi: 10.1136/bmj.c199 [published Online First: 2010/02/09]
9. Moore M, Trill J, Simpson C, et al. Uva-ursi extract and ibuprofen as alternative treatments for uncomplicated urinary tract infection in women (ATAFUTI): a factorial randomized trial. *Clin Microbiol Infect* 2019;25(8):973-80. doi: 10.1016/j.cmi.2019.01.011 [published Online First: 20190125]
10. Vik I, Bollestad M, Grude N, et al. Ibuprofen versus pivmecillinam for uncomplicated urinary tract infection in women-A double-blind, randomized non-inferiority trial. *PLoS Med* 2018;15(5):e1002569. doi: 10.1371/journal.pmed.1002569 [published Online First: 20180515]
11. Wagenlehner FM, Abramov-Sommariva D, Höller M, et al. Non-Antibiotic Herbal Therapy (BNO 1045) versus Antibiotic Therapy (Fosfomycin Trometamol) for the Treatment of Acute Lower Uncomplicated Urinary Tract Infections in Women: A Double-Blind, Parallel-Group, Randomized, Multicentre, Non-Inferiority Phase III Trial. *Urol Int* 2018;101(3):327-36. doi: 10.1159/000493368 [published Online First: 2018/09/20]
12. Carey MR, Vaughn VM, Mann J, et al. Is Non-Steroidal Anti-Inflammatory Therapy Non-Inferior to Antibiotic Therapy in Uncomplicated Urinary Tract Infections: a Systematic Review. *J Gen Intern Med* 2020;35(6):1821-29. doi: 10.1007/s11606-020-05745-x
13. Kaußner Y, Röver C, Heinz J, et al. Reducing antibiotic use in uncomplicated urinary tract infections in adult women: a systematic review and individual participant data meta-analysis. *Clin Microbiol Infect* 2022 doi: https://doi.org/10.1016/j.cmi.2022.06.017
14. Little P, Turner S, Rumsby K, et al. Dipsticks and diagnostic algorithms in urinary tract infection: development and validation, randomised trial, economic analysis, observational cohort and qualitative study. *Health Technol Assess* 2009;13(19):iii-iv, ix-xi, 1-73. doi: 10.3310/hta13190 [published Online First: 2009/04/15]
15. National Institute for Health and Care Excellence (NICE), Urinary tract infection (lower): antimicrobial prescribing; NICE Guideline [NG109]. 2018 . Available from: https://www.nice.org.uk/guidance/ng109 [Accessed June 29, 2021].
16. Scottish Intercollegiate Guidelines Network (SIGN). Management of suspected bacterial lower urinary tract infection in adult women. Edinburgh: SIGN; 2020. (SIGN publication no. 160). [September 2020]. Available from URL: http://www.sign.ac.uk.
17. Schmiemann G, Kniehl E, Gebhardt K, et al. The diagnosis of urinary tract infection: a systematic review. *Dtsch Arztebl Int* 2010;107(21):361-7. doi: 10.3238/arztebl.2010.0361 [published Online First: 2010/06/12]
18. Butler CC, Francis NA, Thomas-Jones E, et al. Point-of-care urine culture for managing urinary tract infection in primary care: a randomised controlled trial of clinical and cost-effectiveness. *Br J Gen Pract* 2018;68(669):e268-e78. doi: 10.3399/bjgp18X695285 [published Online First: 2018/02/28]
19. Holm A, Cordoba G, Møller Sørensen T, et al. Effect of point-of-care susceptibility testing in general practice on appropriate prescription of antibiotics for patients with uncomplicated

For peer review only - http://bmjopen.bmj.com/site/about/guidelines.xhtml

urinary tract infection: a diagnostic randomised controlled trial. *BMJ Open* 2017;7(10):e018028. doi: 10.1136/bmjopen-2017-018028 [published Online First: 20171016]
20. Holm A, Siersma V, Bjerrum L, et al. Availability of point-of-care culture and microscopy in general practice - does it lead to more appropriate use of antibiotics in patients with suspected urinary tract infection? *The European journal of general practice* 2020;26(1):175-81. doi: 10.1080/13814788.2020.1853697
21. Llor C, Moragas A, Alfaro A, et al. Phase-contrast microscopy in the diagnosis of urinary tract infections in the outpatient setting: A diagnostic validity study. *Aten Primaria* 2019;51(7):462-64. doi: 10.1016/j.aprim.2019.02.001 [published Online First: 2019/03/16]
22. Bjerrum L, Grinsted P, Søgaard P. Detection of bacteriuria by microscopy and dipslide culture in general practice. *European Journal of General Practice* 2001;7(2):55-58. doi: 10.3109/13814780109048788
23. Balslev I, Mabeck CE, Thamsborg GM, et al. Urinary infections in general practice. II. Diagnosis. *Ugeskrift for Laeger* 1980;142(26):1661-64.
24. Hallander HO, Kallner A, Lundin A, et al. EVALUATION OF RAPID METHODS FOR THE DETECTION OF BACTERIURIA (SCREENING) IN PRIMARY HEALTH CARE. *Acta Pathologica Microbiologica Scandinavica Series B: Microbiology* 1986;94 B(1-6):39-49. doi: 10.1111/j.1699-0463.1986.tb03018.x
25. Beyer AK, Currea GCC, Holm A. Validity of microscopy for diagnosing urinary tract infection in general practice – a systematic review. *Scand J Prim Health Care* 2019;37(3):373-79. doi: 10.1080/02813432.2019.1639935
26. Rodger M, Ramsay T, Fergusson D. Diagnostic randomized controlled trials: the final frontier. *Trials* 2012;13:137-37. doi: 10.1186/1745-6215-13-137
27. Sanftenberg L, Dreischulte T, Härdtlein A, et al. Process evaluation in practice based research networks: a study protocol for a mixed-methods implementation study. *BMJ Open* 2023;13(7):e065947. doi: 10.1136/bmjopen-2022-065947 [published Online First: 2023/07/13]
28. Altman DG, Bland JM. Treatment allocation by minimisation. *BMJ* 2005;330(7495):843. doi: 10.1136/bmj.330.7495.843
29. Gágyor I, Rentzsch K, Strube-Plaschke S, et al. Psychometric properties of a self-assessment questionnaire concerning symptoms and impairment in urinary tract infections: the UTI-SIQ-8. *BMJ Open* 2021;11(2):e043328. doi: 10.1136/bmjopen-2020-043328 [published Online First: 2021/02/17]
30. ten Cate O. Entrustability of professional activities and competency-based training. *Med Educ* 2005;39(12):1176-7. doi: 10.1111/j.1365-2929.2005.02341.x
31. Damschroder LJ, Reardon CM, Widerquist MAO, et al. The updated Consolidated Framework for Implementation Research based on user feedback. *Implement Sci* 2022;17(1):75. doi: 10.1186/s13012-022-01245-0
32. Deutsche Gesellschaft für Allgemein- und Familienmedizin (DEGAM). S3-Leitlinie: Brennen beim Wasserlassen. AWMF Registernummer: 053-001. 2018 (Available: https://register.awmf.org/assets/guidelines/053-001l_S3_Brennen_beim_Wasserlassen_2018-09-verlaengert_01.pdf, accessed: May 29, 2023).
33. Leitlinienprogramm DGU: Interdisziplinäre S3 Leitlinie: Epidemiologie, Diagnostik, Therapie, Prävention und Management unkomplizierter, bakterieller, ambulant erworbener Harnwegsinfektionen bei erwachsenen Patienten. Langversion 1.1-2, 2017 AWMF Registernummer: 043/044, http://www.awmf.org/uploads/tx_szleitlinien/043-044l_S3_Harnwegsinfektionen pdf (Accessed: November 27, 2022).
34. Schubert S. MIQ 02: Harnwegsinfektionen: Qualitätsstandards in der mikrobiologisch-infektiologischen Diagnostik: Elsevier Health Sciences 2020.
35. Kuckartz U. Qualitative text analysis: A guide to methods, practice and using software: Sage 2014.

For peer review only - http://bmjopen.bmj.com/site/about/guidelines.xhtml

36. WMA Declaration of Helsinki – Ethical Principles for Medical Research Involving Human Subjects (available: https://www.wma.net/policies-post/wma-declaration-of-helsinki-ethical-principles-for-medical-research-involving-human-subjects/).
37. EU General Data Protection Regulation (GDPR): Regulation (EU) 2016/679 of the European Parliament and of the Council of 27 April 2016 on the protection of natural persons with regard to the processing of personal data and on the free movement of such data, and repealing Directive 95/46/EC (General Data Protection Regulation), OJ 2016 L 119/1.

Peter Konstantin Kurotschka

https://orcid.org/0000-0003-3750-6147

Kurotschka_p@ukw.de

Gabor Borgulya: https://orcid.org/0000-0001-6396-6126

Borgulya_g@ukw.de

Eva Bucher

Bucher_e@ukw.de

Isabell Endrich

Endrich_e@ukw.de

Adolfo Figueiras: https://orcid.org/0000-0002-5766-8672

adolfo.figueiras@usc.es

Jochen Gensichen: https://orcid.org/0000-0003-0644-8597

Jochen.Gensichen@med.uni-muenchen.de

Alexander Hapfelmeier: https://orcid.org/0000-0001-6765-6352

Alexander.Hapfelmeier@mri.tum.de

Alastair D Hay: https://orcid.org/0000-0003-3012-375X

Alastair.Hay@bristol.ac.uk

Christian Kretzschmann

Kretzschma_C@ukw.de

For peer review only

For peer review only - http://bmjopen.bmj.com/site/about/guidelines.xhtml

Oliver Kurzai: https://orcid.org/0000-0002-7277-2646

Kurzai_O@ukw.de

Thien-Tri Lam: https://orcid.org/0000-0003-3001-0149

Kurzai_O@ukw.de

Orietta Massidda: https://orcid.org/0000-0001-8823-2372

orietta.massidda@unitn.it

Linda Sanftenberg: https://orcid.org/0000-0002-3174-5297

Linda.Sanftenberg@med.uni-muenchen.de

Guido Schmiemann: https://orcid.org/0000-0002-2846-6561

schmiema@uni-bremen.de

Antonius Schneider: https://orcid.org/0000-0002-2847-8626

antonius.schneider@tum.de

Anne Simmenroth

Simmenroth_A@ukw.de

Stefanie Stark: https://orcid.org/0000-0002-5349-7984

Stefanie.Stark@uk-erlangen.de

Lisette Warkentin: https://orcid.org/0000-0002-6241-6082

Lisette.Warkentin@uk-erlangen.de

Mark H. Ebell: https://orcid.org/0000-0003-3228-2877

ebell@uga.edu

For peer review only

Ildikó Gágyor: https://orcid.org/0000-0002-7974-7603

Gagyor_i@ukw.de

For peer review only

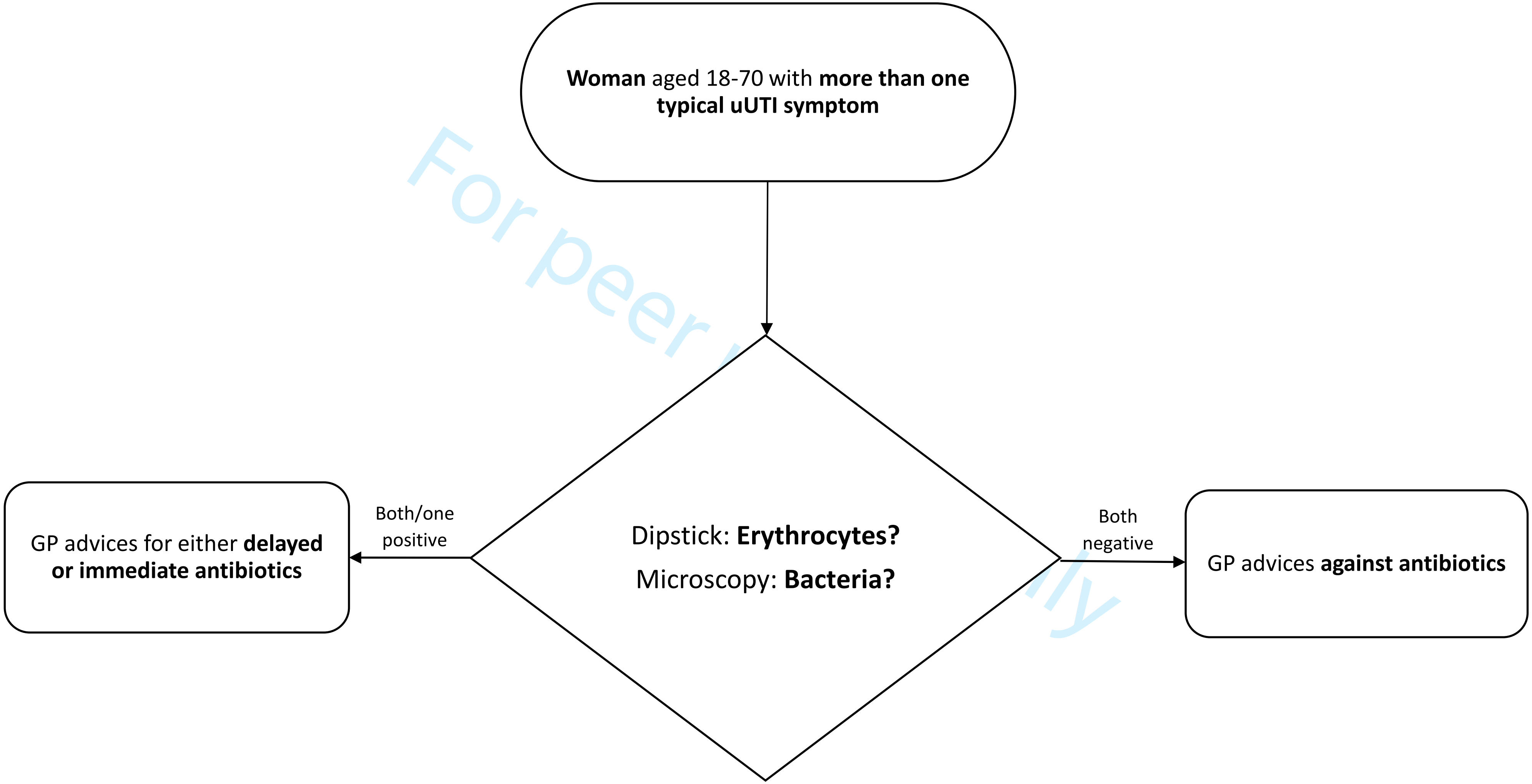

For peer review only

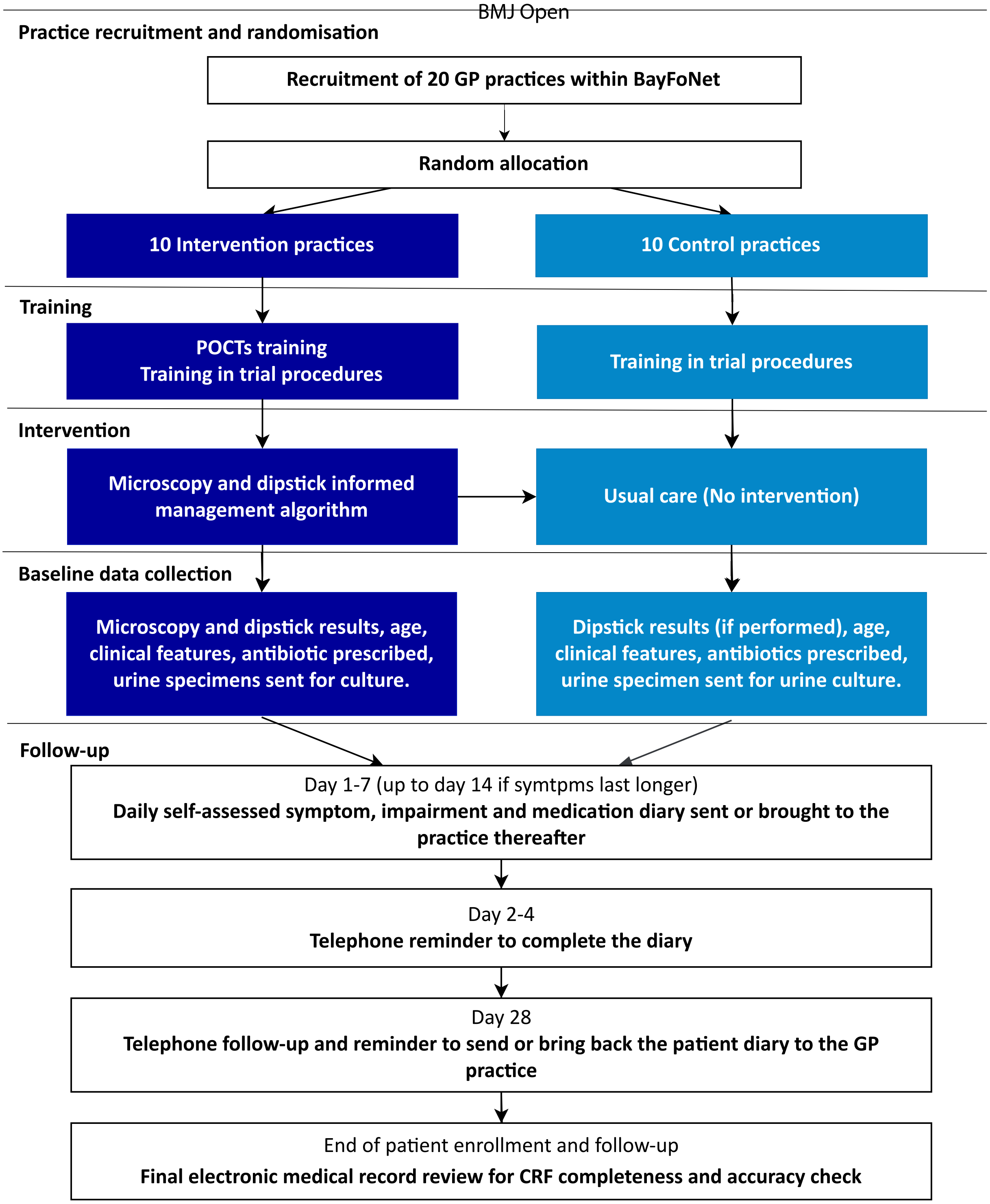
Practice recruitment and randomisation
Recruitment of 20 GP practices within BayFoNet
Random allocation
10 Intervention practices
10 Control practices
Training
POCTs training
Training in trial procedures
Training in trial procedures
Intervention
Microscopy and dipstick informed management algorithm
Usual care (No intervention)
Baseline data collection
Microscopy and dipstick results, age, clinical features, antibiotic prescribed, urine specimens sent for culture.
Dipstick results (if performed), age, clinical features, antibiotics prescribed, urine specimen sent for urine culture.
Follow-up
Day 1-7 (up to day 14 if symtpms last longer)
Daily self-assessed symptom, impairment and medication diary sent or brought to the practice thereafter
Day 2-4
Telephone reminder to complete the diary
Day 28
Telephone follow-up and reminder to send or bring back the patient diary to the GP practice
End of patient enrollment and follow-up
Final electronic medical record review for CRF completeness and accuracy check

SPIRIT
STANDARD PROTOCOL ITEMS: RECOMMENDATIONS FOR INTERVENTIONAL TRIALS

SPIRIT 2013 Checklist: Recommended items to address in a clinical trial protocol and related documents*

Section/item	Item No	Description	Addressed on page number
**Administrative information**			
Title	1	Descriptive title identifying the study design, population, interventions, and, if applicable, trial acronym	Title page 1
Trial registration	2a	Trial identifier and registry name. If not yet registered, name of intended registry	Abstract p. 2
	2b	All items from the World Health Organization Trial Registration Data Set	Abstract p. 2
Protocol version	3	Date and version identifier	Abstract p. 2
Funding	4	Sources and types of financial, material, and other support	P. 14
Roles and responsibilities	5a	Names, affiliations, and roles of protocol contributors	Title page 1
	5b	Name and contact information for the trial sponsor	Title page 1
	5c	Role of study sponsor and funders, if any, in study design; collection, management, analysis, and interpretation of data; writing of the report; and the decision to submit the report for publication, including whether they will have ultimate authority over any of these activities	P. 14
	5d	Composition, roles, and responsibilities of the coordinating centre, steering committee, endpoint adjudication committee, data management team, and other individuals or groups overseeing the trial, if applicable (see Item 21a for data monitoring committee)	P. 13 and 14 where applicable

For peer review only

Introduction

Background and rationale	6a	Description of research question and justification for undertaking the trial, including summary of relevant studies (published and unpublished) examining benefits and harms for each intervention	P. 3 and 4
	6b	Explanation for choice of comparators	P. 9
Objectives	7	Specific objectives or hypotheses	P. 4 and 5
Trial design	8	Description of trial design including type of trial (eg, parallel group, crossover, factorial, single group), allocation ratio, and framework (eg, superiority, equivalence, noninferiority, exploratory)	P. 4

Methods: Participants, interventions, and outcomes

Study setting	9	Description of study settings (eg, community clinic, academic hospital) and list of countries where data will be collected. Reference to where list of study sites can be obtained	P. 5
Eligibility criteria	10	Inclusion and exclusion criteria for participants. If applicable, eligibility criteria for study centres and individuals who will perform the interventions (eg, surgeons, psychotherapists)	P. 6
Interventions	11a	Interventions for each group with sufficient detail to allow replication, including how and when they will be administered	P. 7 - 9
	11b	Criteria for discontinuing or modifying allocated interventions for a given trial participant (eg, drug dose change in response to harms, participant request, or improving/worsening disease)	NA
	11c	Strategies to improve adherence to intervention protocols, and any procedures for monitoring adherence (eg, drug tablet return, laboratory tests)	P. 7 (training)
	11d	Relevant concomitant care and interventions that are permitted or prohibited during the trial	NA
Outcomes	12	Primary, secondary, and other outcomes, including the specific measurement variable (eg, systolic blood pressure), analysis metric (eg, change from baseline, final value, time to event), method of aggregation (eg, median, proportion), and time point for each outcome. Explanation of the clinical relevance of chosen efficacy and harm outcomes is strongly recommended	P. 4 and 5
Participant timeline	13	Time schedule of enrolment, interventions (including any run-ins and washouts), assessments, and visits for participants. A schematic diagram is highly recommended (see Figure)	P. 10, Table 1 and P. 11 Figure 2

For peer review only - http://bmjopen.bmj.com/site/about/guidelines.xhtml

Sample size	14	Estimated number of participants needed to achieve study objectives and how it was determined, including clinical and statistical assumptions supporting any sample size calculations	P. 12 and 13
Recruitment	15	Strategies for achieving adequate participant enrolment to reach target sample size	P. 7 (training)
**Methods: Assignment of interventions (for controlled trials)**			
Allocation:			
Sequence generation	16a	Method of generating the allocation sequence (eg, computer-generated random numbers), and list of any factors for stratification. To reduce predictability of a random sequence, details of any planned restriction (eg, blocking) should be provided in a separate document that is unavailable to those who enrol participants or assign interventions	P. 6
Allocation concealment mechanism	16b	Mechanism of implementing the allocation sequence (eg, central telephone; sequentially numbered, opaque, sealed envelopes), describing any steps to conceal the sequence until interventions are assigned	NA
Implementation	16c	Who will generate the allocation sequence, who will enrol participants, and who will assign participants to interventions	P. 6
Blinding (masking)	17a	Who will be blinded after assignment to interventions (eg, trial participants, care providers, outcome assessors, data analysts), and how	P. 6 (analyst blinding)
	17b	If blinded, circumstances under which unblinding is permissible, and procedure for revealing a participant's allocated intervention during the trial	NA
**Methods: Data collection, management, and analysis**			
Data collection methods	18a	Plans for assessment and collection of outcome, baseline, and other trial data, including any related processes to promote data quality (eg, duplicate measurements, training of assessors) and a description of study instruments (eg, questionnaires, laboratory tests) along with their reliability and validity, if known. Reference to where data collection forms can be found, if not in the protocol	P 9 and 10
	18b	Plans to promote participant retention and complete follow-up, including list of any outcome data to be collected for participants who discontinue or deviate from intervention protocols	P. 9

For peer review only - http://bmjopen.bmj.com/site/about/guidelines.xhtml

Data management	19	Plans for data entry, coding, security, and storage, including any related processes to promote data quality (eg, double data entry; range checks for data values). Reference to where details of data management procedures can be found, if not in the protocol	P.12, 13
Statistical methods	20a	Statistical methods for analysing primary and secondary outcomes. Reference to where other details of the statistical analysis plan can be found, if not in the protocol	P. 12
	20b	Methods for any additional analyses (eg, subgroup and adjusted analyses)	NA (Line 409-410)
	20c	Definition of analysis population relating to protocol non-adherence (eg, as randomised analysis), and any statistical methods to handle missing data (eg, multiple imputation)	P. 12
**Methods: Monitoring**			
Data monitoring	21a	Composition of data monitoring committee (DMC); summary of its role and reporting structure; statement of whether it is independent from the sponsor and competing interests; and reference to where further details about its charter can be found, if not in the protocol. Alternatively, an explanation of why a DMC is not needed	NA (complex intervention, not drug trial)
	21b	Description of any interim analyses and stopping guidelines, including who will have access to these interim results and make the final decision to terminate the trial	NA
Harms	22	Plans for collecting, assessing, reporting, and managing solicited and spontaneously reported adverse events and other unintended effects of trial interventions or trial conduct	NA
Auditing	23	Frequency and procedures for auditing trial conduct, if any, and whether the process will be independent from investigators and the sponsor	NA
**Ethics and dissemination**			
Research ethics approval	24	Plans for seeking research ethics committee/institutional review board (REC/IRB) approval	P. 13
Protocol amendments	25	Plans for communicating important protocol modifications (eg, changes to eligibility criteria, outcomes, analyses) to relevant parties (eg, investigators, REC/IRBs, trial participants, trial registries, journals, regulators)	P. 13

For peer review only - http://bmjopen.bmj.com/site/about/guidelines.xhtml

Consent or assent	26a	Who will obtain informed consent or assent from potential trial participants or authorised surrogates, and how (see Item 32)	P. 6 and 7
	26b	Additional consent provisions for collection and use of participant data and biological specimens in ancillary studies, if applicable	NA
Confidentiality	27	How personal information about potential and enrolled participants will be collected, shared, and maintained in order to protect confidentiality before, during, and after the trial	P. 13
Declaration of interests	28	Financial and other competing interests for principal investigators for the overall trial and each study site	P. 14
Access to data	29	Statement of who will have access to the final trial dataset, and disclosure of contractual agreements that limit such access for investigators	P. 14
Ancillary and post-trial care	30	Provisions, if any, for ancillary and post-trial care, and for compensation to those who suffer harm from trial participation	NA
Dissemination policy	31a	Plans for investigators and sponsor to communicate trial results to participants, healthcare professionals, the public, and other relevant groups (eg, via publication, reporting in results databases, or other data sharing arrangements), including any publication restrictions	P 13
	31b	Authorship eligibility guidelines and any intended use of professional writers	NA
	31c	Plans, if any, for granting public access to the full protocol, participant-level dataset, and statistical code	P 14
**Appendices**			
Informed consent materials	32	Model consent form and other related documentation given to participants and authorised surrogates	Not intended for publication
Biological specimens	33	Plans for collection, laboratory evaluation, and storage of biological specimens for genetic or molecular analysis in the current trial and for future use in ancillary studies, if applicable	P 11-12

*It is strongly recommended that this checklist be read in conjunction with the SPIRIT 2013 Explanation & Elaboration for important clarification on the items. Amendments to the protocol should be tracked and dated. The SPIRIT checklist is copyrighted by the SPIRIT Group under the Creative Commons "Attribution-NonCommercial-NoDerivs 3.0 Unported" license.

BMJ Open

Dipsticks and point-of-care microscopy to reduce antibiotic use in women with an uncomplicated Urinary Tract Infection (MicUTI): Protocol of a randomised controlled pilot trial in primary care

Journal:	*BMJ Open*
Manuscript ID	bmjopen-2023-079345.R1
Article Type:	Protocol
Date Submitted by the Author:	22-Nov-2023

Complete List of Authors:	Kurotschka, Peter Konstantin; Universitätsklinikum Würzburg, Department of General Practice<br>Borgulya, Gábor; University Hospital Würzburg, Department of General Practice<br>Bucher, Eva; University Hospital Würzburg, Department of General Practice<br>Endrich, Isabell; University Hospital Würzburg, Department of General Practice<br>Figueiras, Adolfo; University of Santiago de Compostela, Department of Preventive Medicine and Public Health<br>Gensichen, Jochen; Ludwig Maximilians University Munich, Institute of General Practice and Family Medicine, University Hospital<br>Hay, Alastair; University of Bristol Medical School, Centre for Academic Primary Care<br>Hapfelmeier, Alexander; Technical University of Munich School of Medicine, Department of General Practice and Health Services Research; Technical University of Munich School of Medicine<br>Kretzschmann, Christian; University Hospital Würzburg, Department of General Practice<br>Kurzai, Oliver; University of Würzburg, Institute for Hygiene and Microbiology<br>Lam, Thien-Tri; University of Würzburg, Institute of Hygiene and Microbiology<br>Massidda, Orietta; University of Trento, Department of Cellular, Computational and Integrative Biology<br>Sanftenberg, Linda; University Hospital, Ludwig-Maximilians-Universität, Institute of General Practice and Family Medicine<br>Schmiemann, Guido; University of Bremen, Institute of Public Health and Nursing Research (IPP)<br>Schneider, Antonius; Technical University Munich, Institute of General Practice and Health Services Research<br>Simmenroth, Anne; Julius-Maximilians-Universitat Wurzburg, Department of General Practice<br>Stark, Stefanie; Friedrich-Alexander University Erlangen-Nuremberg, Institute for General Practice and Family Medicine<br>Warkentin, Lisette; Universitätsklinikum Erlangen, Instituteof of General Practice and Family Medicine<br>Ebell, Mark; University of Georgia, Epidemiology and Biostatistics

	Gagyor, Ildiko; Julius-Maximilians-Universitat Wurzburg, Department of General Practice
**Primary Subject Heading**:	General practice / Family practice
Secondary Subject Heading:	Evidence based practice, Infectious diseases
Keywords:	Urinary tract infections < UROLOGY, Randomized Controlled Trial, GENERAL MEDICINE (see Internal Medicine), INFECTIOUS DISEASES

For peer review only - http://bmjopen.bmj.com/site/about/guidelines.xhtml

Dipsticks and point-of-care Microscopy to reduce antibiotic use in women with an uncomplicated Urinary Tract Infection (MicUTI): Protocol of a randomised controlled pilot trial in primary care

Peter K. Kurotschka[1*], Gabor Borgulya[1], Eva Bucher[1], Isabell Endrich[1], Adolfo Figueiras[2], Jochen Gensichen[3], Alaistar D. Hay[4], Alexander Hapfelmeier[5,6], Christian Kretzschmann[1], Oliver Kurzai[7], Thien-Tri Lam[7], Orietta Massidda[8], Linda Sanftenberg[3], Guido Schmiemann[9], Antonius Schneider[4], Anne Simmenroth[1], Stefanie Stark[10], Lisette Warkentin[10], Mark H. Ebell[11], Ildikó Gágyor[1] on behalf of the Bavarian Practice-Based Research Network (BayFoNet)

[1] Department of General Practice, University Hospital Würzburg, Würzburg, Germany

[2] Department of Preventive Medicine and Public Health, University of Santiago de Compostela, Santiago de Compostela, Spain

[3] Institute of General Practice and Family Medicine, University Hospital, Ludwig-Maximilians-Universität München, Munich, Germany

[4] Centre for Academic Primary Care, Bristol Medical School: Population Health Sciences, Department of Community Based Medicine, University of Bristol, Bristol, United Kingdom

[5] Institute of General Practice and Health Services Research, School of Medicine, Technical University of Munich, Munich, Germany

[6] Institute of AI and Informatics in Medicine, School of Medicine, Technical University of Munich, Munich, Germany

[7] Institute for Hygiene and Microbiology, University of Würzburg, Würzburg, Germany

[8] Department of Cellular, Computational and Integrative Biology, University of Trento, Trento, Italy

[9] High-Profile Area of Health Sciences, University of Bremen, Bremen, Germany

[10] Institute of General Practice, Friedrich-Alexander-Universität Erlangen-Nürnberg, University Hospital Erlangen, Erlangen, Germany

[11] Department of Epidemiology and Biostatistics, College of Public Health, University of Georgia, Athens, Georgia, United States

*Author to whom the correspondence should be addressed: (PK Kurotschka) Kurotschka_p@ukw.de

Keywords: Urinary Tract Infections; Women; General Practice; Randomized Controlled Trial; Anti-Infective Agents; Microscopy

For peer review only - http://bmjopen.bmj.com/site/about/guidelines.xhtml

Abstract

Introduction: Uncomplicated urinary tract infections (uUTIs) in women are common infections encountered in primary care. Evidence suggests that rapid point-of-care tests (POCTs) to detect bacteria and erythrocytes in urine at presentation may help primary care clinicians to identify women with uUTIs in whom antibiotics can be withheld without influencing clinical outcomes. This pilot study aims to provide preliminary evidence on whether a POCT informed management of uUTI in women can safely reduce antibiotic use.

Methods and analysis: This is an open-label two-arm parallel cluster-randomised controlled pilot trial. Twenty general practices affiliated with the Bavarian Practice-Based Research Network (BayFoNet) in Germany were randomly assigned to deliver patient management based on POCTs or to provide usual care. POCTs consist of phase-contrast microscopy to detect bacteria and urinary dipsticks to detect erythrocytes in urine samples. In both arms, urine samples will be obtained at presentation for POCTs (intervention arm only) and microbiological analysis. Women will be followed-up for 28 days from enrolment using self-reported symptom diaries, telephone follow-up, and a review of the electronic medical record. Primary outcomes are feasibility of patient enrolment and retention rates per site, which will be summarised by means and SDs, with corresponding confidence and prediction intervals. Secondary outcomes include antibiotic use for UTI at day 28, time to symptom resolution, symptom burden, number of recurrent and upper UTIs and re-consultations, and diagnostic accuracy of POCTs versus. urine culture as the reference standard. These outcomes will be explored at cluster- and individual-levels using descriptive statistics, two-sample hypothesis tests and mixed effects models or generalised estimation equations.

Ethics and dissemination: The University of Würzburg institutional review board approved MicUTI on December 16, 2022 (protocol n. 109/22-sc). Study findings will be disseminated through peer-reviewed publications, conferences, reports addressed to clinicians, and the local citizen´s forums.

Trial registration: ClinicalTrials.gov NCT05667207.

Keywords: Urinary Tract Infections [Mesh]; Cystitis [Mesh]; Female [Mesh]; Humans [Mesh]; Anti-Bacterial Agents [Mesh]; Randomized Controlled Trial [Publication Type]; Pragmatic Clinical Trial [Publication Type]; Point-of-Care Testing [Mesh]; Microscopy [Mesh]; Urinalysis [Mesh]; Bacteriuria [Mesh]; Hematuria [Mesh]

Strenghts and limitations of this study

- This study will inform the design and conduct of future confirmatory RCTs to evaluate the effects of a POCT-guided management strategy on the use of antibiotics in women with suspected uncomplicated UTI in primary care settings.
- The open-label cluster-RCT design implies that participants cannot be identified before randomisation, thus, post-randomisation recruitment may represent a source of selection bias.
- The execution and interpretation of microscopy test results may potentially differ across medical assistants, affecting diagnostic accuracy measurements and treatment outcomes. This will be counteracted by providing all intervention practices with standardised ad-hoc competency-based training.

For peer review only

For peer review only - http://bmjopen.bmj.com/site/about/guidelines.xhtml

INTRODUCTION

Uncomplicated urinary tract infections (UTIs) are a common reason for adult women to consult a healthcare professional and a major driver of antibiotic prescriptions in the community (1). Up to 95% of women presenting to their general practitioner (GP) receive antibiotics, despite uncomplicated UTIs (uUTIs) being self-limiting in up to 50% of cases (1, 2). As antibiotic prescriptions are closely related to the emergence of resistant microorganisms, reducing unnecessary use of these drugs in uUTI management is important to counteract the rise of antimicrobial resistance (AMR) (3-5).

Randomised controlled trials (RCTs) have been conducted to establish whether alternatives to immediate antibiotic treatment, such as delayed prescriptions, herbal formulations, or non-steroidal anti-inflammatory drugs reduce antibiotic use without significant patient harm.(6-11) All strategies were highly effective in reducing antibiotic use over usual care. Nonetheless, if compared with immediate antibiotics, the alternative treatment strategies showed an increased risk of longer symptom duration, higher symptom burden, incomplete recovery, febrile UTI, pyelonephritis and antibiotic use at follow-up (12).

A recent meta-analysis of RCTs confirmed these findings analysing individual participant data of 3524 patients from eight primary care trials (13). In addition, the authors focused on prognostic and moderating factors of treatment effects. They found that, in women treated without antibiotics, incomplete recovery was more likely if, at baseline, erythrocytes were found in urine and women had a positive urine culture (OR 4.68, Bayesian credible interval [CI] 2.07–10.77). If only the urine culture was positive, an incomplete recovery was likely with OR 2.24 (CI 1.00–5.17), while if only the test for erythrocytes was positive the OR was 2.60 (CI 1.086. 32). In contrast, the authors found no significant difference in the likelihood of incomplete recovery between non-antibiotic strategies and immediate antibiotics if both tests were negative (OR 0.82, CI 0.331.95). Moreover, positive urine culture results and erythrocytes in urine were found to be independent predictors of complications such as pyelonephritis, febrile UTI, and of antibiotic use at follow-up (13)-

These findings show a clear benefit of immediate antibiotics in all cases in which the woman´s urine culture is positive, and erythrocytes are found in urine. Simple and affordable tests exist to detect erythrocytes at the point-of-care (POC) rapidly, namely urinary dipsticks (14); however standard urine cultures are not useful for immediate clinical decisions, as they require 48 hours or more to provide definitive results (15-17). This led to studies evaluating portable devices to perform urine culture, which could deliver quicker results than standard cultures and could possibly lead to a more accurate diagnosis than the usual diagnostic approach based only on symptoms and dipstick test results. The multinational POETIC RCT found that POC urine culture and susceptibility testing was not effective at reducing immediate antibiotic prescriptions (18). A Danish RCT found that adding POC susceptibility testing to POC-culture was unlikely to improve the appropriateness of treatment decisions in women with uUTI (19). One explanation for these poor results may be, that POC-cultures require 24 hours to deliver results and, given the prognostic and moderating effects of a

For peer review only - http://bmjopen.bmj.com/site/about/guidelines.xhtml

positive urine culture (i.e. significant bacteriuria) the treatment decision is best to be made immediately during index consultation (13).

To date, urine microscopy is the only rapid POC-test (POCT) able to directly detect bacteria in urine that has been evaluated in a general practice setting. Among the various microscopic methods, phase-contrast microscopy does not require centrifugation or other time-consuming specimen preparation methods, thus being easier to implement in practice than other microscopic approaches (20). Several studies have investigated its diagnostic accuracy in detecting UTI in adult outpatients and have shown promising, although conflicting results. Reported sensitivity varied between 74% and 95%, while specificity ranged between 63% and 97% (21-24). Nevertheless, although a recent systematic review concluded that microscopy “seems to be an accurate, valid and feasible screening-test for bacteriuria in patients with symptoms of UTI in general practice”, it acknowledged that there is still a lack of solid evidence supporting its accuracy to diagnose UTIs.(25)

Diagnostic RCTs have the advantage over traditional diagnostic studies of providing information on test accuracy but also on whether the expected differences in accuracy over usual care are clinically relevant (26). To date, the effects of a diagnostic and treatment strategy guided by POCTs for bacteria and erythrocytes in urine on antibiotic use in women with suspected uUTI have not been studied. This pilot RCT aims to provide information about the feasibility and sample size of a full-scaled diagnostic RCT in general practice and to provide initial data on the effects of a POCT–guided diagnostic and treatment algorithm on antibiotic use. POC-tests to be used are phase-contrast microscopy to detect bacteria and urinary dipsticks to detect erythrocytes in urines.

METHODS AND ANALYSIS

Design

MicUTI (Microscopy in Urinary Tract Infections) is a pragmatic open-label, two-arm parallel cluster randomised pilot trial. General practices are randomly allocated to either the POC-test–based management strategy (intervention) or standard care management. GPs whose practice is allocated to the intervention will have their management guided by phase-contrast microscopy to identify bacteria and urinary dipsticks to identify erythrocytes in urine samples. GPs whose practice is allocated to the control arm will perform usual care, with treatment decisions based mainly on symptoms and dipstick testing for erythrocytes, leukocytes and nitrites. For the trial duration (6 months), practices are asked to systematically recruit women aged 18 to 70 years with symptoms suggesting a uUTI (dysuria, frequency/urgency of micturition, nocturia, with or without lower abdominal pain) to be included in the trial.

Objectives

Primary objective

The primary objective of this study is to assess the feasibility of a full-scaled diagnostic RCT. Primary endpoints are patient recruitment efficacy and percentage of retention in the trial. Recruitment efficacy will be calculated as the number of participants enrolled per site over

the six months of trial duration, and retention as the percentage of complete follow-ups per site over 28 days. A confirmatory study without major design changes will be considered feasible if study sites recruit 10 patients each and patients lost to follow-up do not exceed 20%.

Secondary Objectives

The secondary objectives of the MicUTI trial are to compare the intervention and control practices with regard to the following endpoints:

1. Total antibiotic use: number of antibiotic prescriptions per patient with UTI within 28 days
2. Defined daily doses of the prescribed antibiotics per patient with UTI within 28 days
3. Inappropriate antibiotic use: the percentage of patients with symptoms of UTI who were prescribed antibiotics among those with negative urine culture findings
4. Number of immediate and delayed antibiotic prescriptions for uUTI at initial consultation
5. Number of early relapses of UTI (days 0–14)
6. Number of recurrent UTIs (days 15–28)
7. Number of upper UTIs within 28 days
8. Number of consultations due to UTI (or symptoms of UTI) within 28 days
9. Time to complete symptom resolution defined as a maximum of 1 point in each of the UTI–SIQ–8 items (27)
10. Total symptom burden on days 0–7 (area under the curve of the UTISIQ8 total symptom score)
11. Diagnostic accuracy of microscopy with or without concomitant dipstick test compared with the reference standard test (urine culture)

A further objective of this study is to evaluate the feasibility and acceptability of POC-microscopy to detect bacteria in urine and the training sessions addressed to medical assistants in the intervention arm practices.

Setting

The MicUTI study is conducted in a primary care research network based in the German Federal State of Bavaria, the Bavarian Practice-Based Research Network (BayFoNet). Three out of the five university departments part of the BayFoNet participate in MicUTI. These are the Department of General Practice of the University Hospital Würzburg (hereafter abbreviated as UKW), which is the trial coordinating centre, the Institute of General Practice of the Friedrich-Alexander Universität Erlangen-Nürnberg (hereafter abbreviated as FAU) and the Institute of General Practice and Family Medicine of the Ludwig-Maximilians University Hospital Munich (hereafter abbreviated as LMU), who collaborate in the recruitment of study sites and data collection. An accompanying mixed-methods process evaluation assesses the implementation of this trial into the BayFoNet and further investigates its feasibility and

For peer review only - http://bmjopen.bmj.com/site/about/guidelines.xhtml

acceptability to general practice teams. Details of the process evaluation study are published elsewhere (28).

Recruitment of study sites

The UKW and the FAU will 20 general practices located in the Federal State of Bavaria (Würzburg and Erlangen and surrounding areas) to participate in the trial through postal invitations, institutional advertisements such as newsletters and website announcements, and public conferences with GP practice teams.

Randomisation of study sites

General practices participating in the trial (hereafter: study sites) will be asked to provide the number of consulting patients over the last year to determine practice size. Study sites of larger size are foreseeably able to recruit more patients for the duration of the trial, so that practice size will be balanced between the two arms (each with ten practices) using minimization (29). The minimisation algorithm is centrally programmed in Python by the study statistician. To mask the trial statistician (GB) to intervention/control assignments, the minimisation algorithm will be executed by the trial´s statistical consultant based at the Technical University of Munich (AH), who is not involved in the conduction of the trial. Intervention/control assignments of practices will be communicated to the UKW and the FAU, who then will communicate their allocation to the study sites. To minimise selection bias, study sites were identified for inclusion in the trial prior to randomisation.

Patient recruitment/enrolment

Identification of women eligible for inclusion

At presentation, at all study sites, medical assistants will consecutively approach all adult women presenting with two or more symptoms suggestive of acute uUTI (dysuria, frequency, urgency, nocturia, lower abdominal pain) to identify trial participants. To ensure consecutive enrollment and control for selection bias, all potential participants will be approached and listed in paper-based anonymous patient pre-screening logs at each study site. Interested women will be asked to complete the validated symptom questionnaire UTI-SIQ-8 (27) and will be provided with a comprehensive patient information leaflet which details trial procedures in lay language.

The GP will exclude from participation all women with one or more of the following:

- Signs of a complicated UTI (anamnesis of fever, chills or flank pain)
- Clinically relevant immunosuppression (i.e., current use of any immunosuppressive therapy, congenital or acquired disorders of immunity)
- Acute or chronic functional or anatomical variations in the urinary tract except for chronic kidney failure with eGFR > 45 ml/min
- Permanent bladder catheter or use of bladder catheter within the past two weeks
- UTI within the past two weeks
- Use of any antibiotic within the past two weeks

For peer review only - http://bmjopen.bmj.com/site/about/guidelines.xhtml

- Accommodation in a nursing home or hospital stay within the past two weeks
- Severe neurologic, psychiatric illness, severe dementia or severe substance use disorder
- Other severe diseases
- Being unable to understand the informed consent or to complete the patient diary
- Known pregnancy

Women who consent will be asked to sign an informed consent form, which also contains data protection regulations.

Intervention arm

GPs whose practice is allocated to the intervention will have their management guided by POCTs, namely phase-contrast microscopy and urinary dipsticks, for all patients consenting to participation.

Point-of-care tests

Medical assistants will perform microscopy using phase-contrast microscopes ("Primostar", Carl Zeiss Suzhou Co.®, Suzhou, China) to examine 7 µL of clean-catch mid-stream urine (MSU) without prior centrifugation at 400x magnification in a precision counting chamber (Fast-Read® 102 slides, Biosigma s.r.l., Cona (VE), Italy) to detect bacteria. A test will be considered positive if more than a few bacteria of the same shape (rod or cocci) or if many bacteria of different shapes (rod and cocci) are detected per high power field (30).

Urinary dipstick analysis will be performed using COMBUR5-Test® (Roche Diagnostics GmBH, Mannheim, Germany) to examine 50 mL MSU to identify erythrocytes. In accordance with the manufacturer instructions, the COMBUR5®-test strip will be dipped in the urine sample for about one second, wiped against the rim of the vessel to remove excess urine and read manually after 60 seconds by comparing the colour of the detection pad of the strip with the colour scale on the test strip vial. A positive test result is defined as the color change of the strip corresponding to 1+ erythrocytes or greater.

All the analyses will be conducted immediately after the MSU specimen collection at each study site.

Training of study sites in trial procedures

Medical assistants will be provided with ad-hoc training to approach potentially eligible women consecutively, and to perform POCTs according to the study protocol by dedicated staff. Training will be delivered face-to-face prior to patient recruitment over a three-hour session (baseline session), as well as three months after the start of patient recruitment (refresher session), following the principles of competency-based medical education (31). The baseline training session will entail the following: (1) Guideline knowledge on UTI in women to allow medical assistants to identify potentially eligible women in each study site; (2) POCT training to perform and interpret the results of phase-contrast microscopy and urinary

For peer review only - http://bmjopen.bmj.com/site/about/guidelines.xhtml

For peer review only

dipsticks according to the study protocol. The knowledge and skills gained, and their retention, as well as the feasibility and the acceptability of the training sessions and the use of phase-contrast microscopes in daily practice, will be assessed through a questionnaire addressed to medical assistants before, immediately after, and three months after the training session, and through face-to-face semi-structured interviews, three months after the training session. Interviews will be conducted following a pre-planned interview-guide informed by the Consolidated Framework for Implementation Research (32). Practical tests at the microscope directly after the training session and after three months will assess each medical assistant's acquisition and retention of practical skills.

Point-of-care test guided treatment algorithm

Based on the prognostic and moderating effects on treatment outcomes of erythrocytes and bacteria in urine (13), GPs will be encouraged to apply the following treatment algorithm (Figure 1) to consenting women, taking their preferences into account:

1. If POCTs are positive for bacteria by microscopy and/or for erythrocytes by dipsticks, the GP issues, at their own clinical judgement, a delayed or immediate prescription for an antibiotic. In the MicUTI intervention, a delayed prescription can be issued by GPs, at their discretion, in either way: the prescription is handed out to the patient with the advice to redeem it only when symptoms do not improve or worsen in 48 hours, or it is withheld in the GP practice. It can be handed out if the patient present again with complaints.
2. If POCTs are negative for bacteria and erythrocytes, the GP advises for self-help remedies according to national guidelines and to do without antibiotics (33, 34).

Insert figure 1

Data collection

Baseline

Baseline data will be collected during routine consultation. The medical assistant records microscopy and dipstick test results, clinical features, and the GP´s management decisions, including antibiotics prescribed on a baseline paper-based CRF. In addition, the medical assistant inoculates a dipslide (see paragraph “microbiological analyses”) to be send via post on the same day to the central research laboratory based at the Institute of Hygiene and Microbiology of the Julius-Maximilians-University of Würzburg (IHM) for microbiological analysis.

Follow-up

Follow-up data will be collected through a self-directed patient diary, follow-up telephone calls at day 28 from inclusion, and follow-up in the GP practice in case of re-consultation. In addition, an electronic medical records (EMR) review will also be performed to double-check for missing follow-up data.

For peer review only - http://bmjopen.bmj.com/site/about/guidelines.xhtml

Patient diary. Women will be asked to rate their daily symptoms and impairment due to UTI on a scale from 1 (no symptoms/impairment at all) to 5 (very strong symptoms/impairment) and to write down the antibiotics taken in a diary for at least seven days (or up to 14 days if symptoms last longer) (27). Enough space in the diary is left for women to take extra notes. They will be asked to return the completed diary to their GP practice using a pre-paid envelope or in person. Research nurses at the FAU and the LMU will perform telephone calls on day 2-4 from inclusion to remind enrolled women to fill out and return the diary.

Follow-up telephone calls. Calls will be performed by research nurses at the FAU and the LMU on day 28 from inclusion to collect data on symptoms, antibiotic use due to UTI, recurrences, and re-consultations with UTI symptoms.

Follow-up in the GP practice. Every time a woman re-consults for UTI within 28 days, the GP records clinical features, management decisions, and antibiotics prescribed on follow-up paper-based CRFs.

Electronic medical records review. Researchers from the UKW and the FAU will perform outreach visits at the end of the patient recruitment (6 months) in each of the study sites. Paper-based CRFs will be collected, and data will be controlled for completeness. To double-check data entries for accuracy and completeness, EMRs will be enquired on re-consultations with UTI symptoms, recurrences, antibiotics prescribed due to UTI, and upper UTI until day 28 from inclusion.

Control arm–usual care

Practices in the control arm will not necessarily have their management guided by POCTs. They will perform usual care. The treatment decision is usually based on symptoms. Dipsticks for detecting erythrocytes, leucocyte esterase or nitrites can be added in case of diagnostic uncertainty (33). Procedures are the same as those outlined above, except for POCTS: microscopy only pertains to the intervention arm.

Table 1 and Figure 2 summarise trial procedures.

Table 1. Schedule of MicUTI trial procedures according to the Standard Protocol Items: Recommendations for Interventional Trials (SPIRIT) guideline (35).

**TIMEPOINT (Day 0 = patient enrolment)** ***Procedures***	**< 0**	**Day 0** Baseline	**Day 1-7**	**Day 28**	**End of patient enrollment**[a]
**ENROLMENT:**					
Practice recruitment	X				
Randomisation	X				
Allocation	X				
Training	X				
Screening for potential inclusion		X			
Formal eligibility assessment		X			

For peer review only - http://bmjopen.bmj.com/site/about/guidelines.xhtml

**INTERVENTION:**					
Baseline consultation[1]		X			
POCTs[2]		X			
Urine sent to laboratory[1]		X			
**ASSESSMENTS:**					
Practice size	X				
Paper based-CRF[3]		X			
Patient diary[4]			X		
Follow-up telephone calls				X	
EMR review[5]					X

[a] End of patient enrollment in each practice

[1] Applies to intervention and control arm practices

[2] Applies only to intervention arm practices

[3] In case of re-consultation for UTI on days 1–28, paper-based follow–up CRFs are used

[4] Follow-up on days 1–7 or up to day 14 if symptoms last longer or recur.

[5] Follow-up on days 1–28 (CRF completeness and accuracy check)

Insert Figure 2

Microbiological analyses

For cultural urine diagnostics, at each study site, medical assistants immerse a dipslide, i.e. a culture medium carrier (Uricult® Plus, Roche Diagnostics GmbH, Mannheim, Germany) into the test urine until the agar surface is completely covered. After removal from the urine, the carrier is placed into a sterile transport tube for transportation via post to the IHM. Upon arrival, the carrier is incubated at 35 ± 2°C for 16 to 24 h. If bacterial growth is detected, the subsequent analysis is carried out according to the established diagnostic procedures of the accredited medical laboratory. Briefly, the analysis includes determination of the overall bacterial count on the Cystine Lactose Electrolyte Deficient–agar (CLED) plates, and those of Gram-negative bacteria (on MacConkey agar plates) and of *Enterococcus spp.* (on the *Enterococcus* agar plates). Bacteria are subcultured on Columbia Sheep Blood agar plates and MacConkey agar plates to be subsequently identified by mass spectrometry (VITEK MS, BioMérieux, Marcy-l'Etoile, France). Susceptibility testing of pathogenic bacteria is achieved using VITEK 2 (BioMérieux, Marcy-l'Etoile, France) and interpreted according to the EUCAST criteria (36).

To define a significant growth in the MSU specimens, the criteria specified in the updated 2020 German infectious disease and microbiology laboratory quality standards will be used.(30)

Statistical analyses

Descriptive statistics will be calculated for all variables per site and as summary measures across sites. Continuous and count variables will be summarised by mean and SD, or median and IQR in case of skewed data. Binary and categorical variables will be summarised through counts and percentages. Confidence intervals and prediction intervals will be calculated for

For peer review only

For peer review only - http://bmjopen.bmj.com/site/about/guidelines.xhtml

the two feasibility endpoints (recruitment efficacy and percentage of retention in the trial) in addition.

We acknowledge that this feasibility RCT is not powered to detect any statistically significant effects. Nevertheless, explorative analyses will be undertaken at the cluster-level and the individual-level to evaluate the effects of the intervention on the secondary outcome variables using two-sample hypothesis tests, random effects models and generalised estimation equations, as appropriate. Alongside point-estimates, p-values and 95% confidence intervals will be reported.

Missing data will be explored to understand why they are unavailable; when missing data are due to incomplete follow-up survival or censored regression analyses will be considered, otherwise multiple imputation with chained equations or Bayesian imputation will be applied. The intraclass correlation coefficient will be calculated for the secondary endpoints to inform subsequent sample size and power calculations.

All the analyses will be undertaken using statistical software packages Stan (37), R, and STATA (StataCorp. College Station, TX: StataCorp LLC).

Qualitative data analysis of interviews addressed to medical assistants

All interviews will be de-identified, transcribed verbatim, and analysed by at least two researchers inductively via content-analysis inspired by Kuckartz (38). The analysts perform first line-by-line coding after extensive reading of the interviews (familiarisation). While iteratively refining the code labels, they are grouped to generate categories and subcategories. In subsequent iterations of the process, categories and subcategories are refined in light of the research question and are summarised in a final report of the analyses.

Sample size considerations

An ad hoc analysis undertaken on claims data provided by the Bavarian health insurance union (Kassenärztliche Vereinigung) covering all diagnosed UTIs in ambulatory care from 2015 to 2019 showed that each GP had an average of 60 encounters for uUTI in women aged 18-70 per year, with only slight seasonal fluctuations (data not shown). Considering that some of these encounters occur in out-of-hours care and that not all of the patients presenting with uUTI are going to be eligible, we expect that 200 patients in both arms (i. e. 100 patients per arm = an average of ten enrolled patients in each of the 20 clusters) is a reasonably achievable sample within six months of trial duration.

Assuming that the percentage of complete follow-up is 75% in the population the patients are sampled from, a simulation study showed that the 200 patients of the feasibility trial enable the estimation of the percentage of complete follow-up with a 95% CI of width of less than 13% with a likelihood of 0.90.

Patient and public involvement

For peer review only - http://bmjopen.bmj.com/site/about/guidelines.xhtml

A citizens' forum was established within the Bavarian Practice Based Research Network (BayFoNet) framework. Invited are all citizens who are interested in contributing to research projects of the Department of General Practice (University Hospital Würzburg). All important steps that are necessary for different projects and studies are discussed with the participants. The MicUTI study has been discussed with the citizens twice so far. After the presentation of the study, the participants discussed whether therapy for uUTI without antibiotics would be conceivable. In addition, the members gave feedback on the comprehensibility and clarity of the patient diary and the patient information.
Furthermore, feedback was given in the following meeting of the citizens' forum on how the citizens' comments were incorporated into the further work.

Trial status

Patient recruitment started in June 2023 and is ongoing. Planned end of patient recruitment is in April 2024.

ETHICS AND DISSEMINATION

The study will be conducted in accordance to the Declaration of Helsinki in its current version (39). The University of Würzburg institutional review board approved MicUTI on December 16, 2022 (protocol n. 109/22-sc). The recording, disclosure, storage and analysis of personal data within this clinical trial will take place by the legal provisions of the EU General Data Protection Regulation (GDPR). To protect confidentiality, all data will be collected and stored psudonymised. Pseudonymisation lists will be stored in a secure place at each study sites separately from CRFs (40). Details of procedures to ensure confidentiality are provided in the supplementary file.
A trial steering committee is established. The committee is composed by the principal investigator (IG), the study coordinator (PK) and by internationally renowned experts in the implementation and conduction of pragmatic RCTs in primary care and research methodology (AH, AF, AH, GS, MHE). The committee provides an overall methodological supervision of the study and safeguards the interest of study participants. Any of its members will be informed of amendments to the protocol.

The implementation of MicUTI will generate the needed knowledge to plan a confirmatory, full-scaled trial, intended to reduce the clinical equipoise about rapid POCTs used to inform the clinical management of uUTIs in general practice. Study results will be disseminated through peer-reviewed publications, academic conferences, a report addresses to participating GPs and through a summary report in lay language addressed to patients and the public.

Author contributions. PK and IG conceived the study and are primarily responsible for the study design. GB, together with PK, planned the randomisation, the statistical analyses and the sample size. PK wrote the protocol consulting IG, AH, AF, GS, GB, ADH, AS, ASc and MHE (trial design), OM, TTL, and OK (point-of-care tests and

For peer review only - http://bmjopen.bmj.com/site/about/guidelines.xhtml

microbiological analyses), IG and CK (data protection and PPI), EB, IE, StS, LW, LS, and JG (local implementation, data collection). All authors read and approved the final version of the manuscript.

Funding. The present study is funded by the German Federal Ministry of Education and Research (BMBF) grant Nr. 01GK1903A "Development of a sustainable structure for practice-based research networks for the strengthening of general practice".
The funder had no role or authority in the design, or in the writing of the protocol. Moreover, the funder will have no role or authority in data collection, data management, data analysis, interpretation, reporting, writing up or publication of findings.

Competing interests. None declared.

Acknowledgements. We are grateful for all participating general practices. A particular thank goes to Waltraud Flederer, Merle Klanke, Maike Ermster, Kathrin Lasher, Anna-Lena Schnaidt and Petra Hagenbusch for their help in the field implementation. We also thank Alice Serafini, MD, for her genuine advices in the planning of the field implementation.
PK and EB, as PhD and MD candidates respectively, are supported by funds of the Bavarian State Ministry of Science and Arts and the University of Würzburg to the Graduate School of Life Sciences, University of Würzburg.
Bavarian Practice-Based Research Network (BayFoNet) members include, in alphabetical order: Andrea Baumgärtel, Melanie Bößenecker, Tobias Dreischulte, Stefanie Eck, Ildikó Gágyor, Jochen Gensichen, Alexander Hapfelmeier, Susann Hueber, Merle Klanke, Christian Kretzschmann, Thomas Kühlein, Peter K. Kurotschka, Kathrin Lasher, Klaus Linde, Klara Lorenz-Dant, Marco Roos, Linda Sanftenberg, Antonius Schneider, Stefanie Stark, Til Uebel, Fabian Walter.

References

1. Butler CC, Hawking MK, Quigley A, McNulty CA. Incidence, severity, help seeking, and management of uncomplicated urinary tract infection: a population-based survey. Br J Gen Pract. 2015;65(639):e702-7.
2. Hoffmann T, Peiris R, Mar CD, Cleo G, Glasziou P. Natural history of uncomplicated urinary tract infection without antibiotics: a systematic review. Br J Gen Pract. 2020;70(699):e714-e22.
3. Costelloe C, Metcalfe C, Lovering A, Mant D, Hay AD. Effect of antibiotic prescribing in primary care on antimicrobial resistance in individual patients: systematic review and meta-analysis. BMJ. 2010;340:c2096.
4. Laxminarayan R. The overlooked pandemic of antimicrobial resistance. The Lancet. 2022;399(10325):606-7.
5. Murray CJL, Ikuta KS, Sharara F, Swetschinski L, Robles Aguilar G, Gray A, et al. Global burden of bacterial antimicrobial resistance in 2019: a systematic analysis. The Lancet. 2022;399(10325):629-55.
6. Gágyor I, Bleidorn J, Kochen MM, Schmiemann G, Wegscheider K, Hummers-Pradier E. Ibuprofen versus fosfomycin for uncomplicated urinary tract infection in women: randomised controlled trial. BMJ (Clinical research ed). 2015;351:h6544.
7. Gágyor I, Hummers E, Schmiemann G, Friede T, Pfeiffer S, Afshar K, et al. Herbal treatment with uva ursi extract versus fosfomycin in women with uncomplicated urinary tract infection in primary care: a randomized controlled trial. Clin Microbiol Infect. 2021;27(10):1441-7.
8. Little P, Moore MV, Turner S, Rumsby K, Warner G, Lowes JA, et al. Effectiveness of five different approaches in management of urinary tract infection: randomised controlled trial. Bmj. 2010;340:c199.

For peer review only - http://bmjopen.bmj.com/site/about/guidelines.xhtml

9. Moore M, Trill J, Simpson C, Webley F, Radford M, Stanton L, et al. Uva-ursi extract and ibuprofen as alternative treatments for uncomplicated urinary tract infection in women (ATAFUTI): a factorial randomized trial. Clin Microbiol Infect. 2019;25(8):973-80.
10. Vik I, Bollestad M, Grude N, Bærheim A, Damsgaard E, Neumark T, et al. Ibuprofen versus pivmecillinam for uncomplicated urinary tract infection in women-A double-blind, randomized non-inferiority trial. PLoS Med. 2018;15(5):e1002569.
11. Wagenlehner FM, Abramov-Sommariva D, Höller M, Steindl H, Naber KG. Non-Antibiotic Herbal Therapy (BNO 1045) versus Antibiotic Therapy (Fosfomycin Trometamol) for the Treatment of Acute Lower Uncomplicated Urinary Tract Infections in Women: A Double-Blind, Parallel-Group, Randomized, Multicentre, Non-Inferiority Phase III Trial. Urol Int. 2018;101(3):327-36.
12. Carey MR, Vaughn VM, Mann J, Townsend W, Chopra V, Patel PK. Is Non-Steroidal Anti-Inflammatory Therapy Non-Inferior to Antibiotic Therapy in Uncomplicated Urinary Tract Infections: a Systematic Review. J Gen Intern Med. 2020;35(6):1821-9.
13. Kaußner Y, Röver C, Heinz J, Hummers E, Debray TPA, Hay AD, et al. Reducing antibiotic use in uncomplicated urinary tract infections in adult women: a systematic review and individual participant data meta-analysis. Clin Microbiol Infect. 2022.
14. Little P, Turner S, Rumsby K, Warner G, Moore M, Lowes JA, et al. Dipsticks and diagnostic algorithms in urinary tract infection: development and validation, randomised trial, economic analysis, observational cohort and qualitative study. Health Technol Assess. 2009;13(19):iii-iv, ix-xi, 1-73.
15. National Institute for Health and Care Excellence (NICE), Urinary tract infection (lower): antimicrobial prescribing; NICE Guideline [NG109]. 2018 . Available from: https://www.nice.org.uk/guidance/ng109 [Accessed June 29, 2021].
16. Scottish Intercollegiate Guidelines Network (SIGN). Management of suspected bacterial lower urinary tract infection in adult women. Edinburgh: SIGN; 2020. (SIGN publication no. 160). [September 2020]. Available from URL: http://www.sign.ac.uk.
17. Schmiemann G, Kniehl E, Gebhardt K, Matejczyk MM, Hummers-Pradier E. The diagnosis of urinary tract infection: a systematic review. Dtsch Arztebl Int. 2010;107(21):361-7.
18. Butler CC, Francis NA, Thomas-Jones E, Longo M, Wootton M, Llor C, et al. Point-of-care urine culture for managing urinary tract infection in primary care: a randomised controlled trial of clinical and cost-effectiveness. Br J Gen Pract. 2018;68(669):e268-e78.
19. Holm A, Cordoba G, Møller Sørensen T, Rem Jessen L, Frimodt-Møller N, Siersma V, et al. Effect of point-of-care susceptibility testing in general practice on appropriate prescription of antibiotics for patients with uncomplicated urinary tract infection: a diagnostic randomised controlled trial. BMJ Open. 2017;7(10):e018028.
20. Holm A, Siersma V, Bjerrum L, Cordoba G. Availability of point-of-care culture and microscopy in general practice - does it lead to more appropriate use of antibiotics in patients with suspected urinary tract infection? The European journal of general practice. 2020;26(1):175-81.
21. Llor C, Moragas A, Alfaro A, Sierra N. Phase-contrast microscopy in the diagnosis of urinary tract infections in the outpatient setting: A diagnostic validity study. Aten Primaria. 2019;51(7):462-4.
22. Bjerrum L, Grinsted P, Søgaard P. Detection of bacteriuria by microscopy and dipslide culture in general practice. European Journal of General Practice. 2001;7(2):55-8.
23. Balslev I, Mabeck CE, Thamsborg GM, Vejlsgaard R. Urinary infections in general practice. II. Diagnosis. Ugeskrift for Laeger. 1980;142(26):1661-4.
24. Hallander HO, Kallner A, Lundin A, ÖSterberg E. EVALUATION OF RAPID METHODS FOR THE DETECTION OF BACTERIURIA (SCREENING) IN PRIMARY HEALTH CARE. Acta Pathologica Microbiologica Scandinavica Series B: Microbiology. 1986;94 B(1-6):39-49.
25. Beyer AK, Currea GCC, Holm A. Validity of microscopy for diagnosing urinary tract infection in general practice – a systematic review. Scandinavian Journal of Primary Health Care. 2019;37(3):373-9.
26. Rodger M, Ramsay T, Fergusson D. Diagnostic randomized controlled trials: the final frontier. Trials. 2012;13:137-.

For peer review only - http://bmjopen.bmj.com/site/about/guidelines.xhtml

27. Gágyor I, Rentzsch K, Strube-Plaschke S, Himmel W. Psychometric properties of a self-assessment questionnaire concerning symptoms and impairment in urinary tract infections: the UTI-SIQ-8. BMJ Open. 2021;11(2):e043328.
28. Sanftenberg L, Dreischulte T, Härdtlein A, Kosub H, Gagyor I, Kurotschka PK, et al. Process evaluation in practice based research networks: a study protocol for a mixed-methods implementation study. BMJ Open. 2023;13(7):e065947.
29. Altman DG, Bland JM. Treatment allocation by minimisation. BMJ. 2005;330(7495):843.
30. Schubert S. MIQ 02: Harnwegsinfektionen: Qualitätsstandards in der mikrobiologisch-infektiologischen Diagnostik: Elsevier Health Sciences; 2020.
31. ten Cate O. Entrustability of professional activities and competency-based training. Med Educ. 2005;39(12):1176-7.
32. Damschroder LJ, Reardon CM, Widerquist MAO, Lowery J. The updated Consolidated Framework for Implementation Research based on user feedback. Implement Sci. 2022;17(1):75.
33. Deutsche Gesellschaft für Allgemein- und Familienmedizin (DEGAM). S3-Leitlinie: Brennen beim Wasserlassen. AWMF Registernummer: 053-001. 2018 (Available: https://register.awmf.org/assets/guidelines/053-001l_S3_Brennen_beim_Wasserlassen_2018-09-verlaengert_01.pdf, accessed: May 29, 2023).
34. Leitlinienprogramm DGU: Interdisziplinäre S3 Leitlinie: Epidemiologie, Diagnostik, Therapie, Prävention und Management unkomplizierter, bakterieller, ambulant erworbener Harnwegsinfektionen bei erwachsenen Patienten. Langversion 1.1-2, 2017 AWMF Registernummer: 043/044, http://www.awmf.org/uploads/tx_szleitlinien/043- 044l_S3_Harnwegsinfektionen pdf (Accessed: November 27, 2022).
35. Chan A-W, Tetzlaff JM, Altman DG, Laupacis A, Gøtzsche PC, Krleža-Jerić K, et al. SPIRIT 2013 Statement: Defining Standard Protocol Items for Clinical Trials. Annals of Internal Medicine. 2013;158(3):200-7.
36. EUCAST Expert rules in antimicrobial susceptibility testing, version 1, April 2008

Available: https://www.eucast.org/fileadmin/src/media/PDFs/4ESCMID_Library/3Publications/EUCAST_Documents/Other_Documents/EUCAST_Expert_rules_final_April_20080407.pdf Accessed: May 12, 2022.
37. Stan Development Team. 2022. Stan Modeling Language Users Guide and Reference Manual, 2.33. https://mc-stan.org.
38. Kuckartz U. Qualitative text analysis: A guide to methods, practice and using software: Sage; 2014.
39. WMA Declaration of Helsinki – Ethical Principles for Medical Research Involving Human Subjects (available: https://www.wma.net/policies-post/wma-declaration-of-helsinki-ethical-principles-for-medical-research-involving-human-subjects/).
40. EU General Data Protection Regulation (GDPR): Regulation (EU) 2016/679 of the European Parliament and of the Council of 27 April 2016 on the protection of natural persons with regard to the processing of personal data and on the free movement of such data, and repealing Directive 95/46/EC (General Data Protection Regulation), OJ 2016 L 119/1.

Figure legends

Figure 1. Treatment algorithm in the intervention arm

Abbreviations. uUTI = uncomplicated urinary tract infection; GP = general practitioner

Figure 2. Flow diagram of the MicUTI trial procedures

For peer review only - http://bmjopen.bmj.com/site/about/guidelines.xhtml

Abbreviations. POCTs = Point-of-Care tests; GP = general practitioner; CRF = case report form

Peter K. Kurotschka

https://orcid.org/0000-0003-3750-6147

Kurotschka_p@ukw.de

Gabor Borgulya: https://orcid.org/0000-0001-6396-6126

Borgulya_g@ukw.de

Eva Bucher

Bucher_e@ukw.de

Isabell Endrich

Endrich_i@ukw.de

Adolfo Figueiras: https://orcid.org/0000-0002-5766-8672

adolfo.figueiras@usc.es

Jochen Gensichen: https://orcid.org/0000-0003-0644-8597

Jochen.Gensichen@med.uni-muenchen.de

Alexander Hapfelmeier: https://orcid.org/0000-0001-6765-6352

Alexander.Hapfelmeier@mri.tum.de

Alastair D Hay: https://orcid.org/0000-0003-3012-375X

Alastair.Hay@bristol.ac.uk

Christian Kretzschmann

Kretzschma_C@ukw.de

For peer review only - http://bmjopen.bmj.com/site/about/guidelines.xhtml

Oliver Kurzai: https://orcid.org/0000-0002-7277-2646

Kurzai_O@ukw.de

Thien-Tri Lam: https://orcid.org/0000-0003-3001-0149

Kurzai_O@ukw.de

Orietta Massidda: https://orcid.org/0000-0001-8823-2372

orietta.massidda@unitn.it

Linda Sanftenberg: https://orcid.org/0000-0002-3174-5297

Linda.Sanftenberg@med.uni-muenchen.de

Guido Schmiemann: https://orcid.org/0000-0002-2846-6561

schmiema@uni-bremen.de

Antonius Schneider: https://orcid.org/0000-0002-2847-8626

antonius.schneider@tum.de

Anne Simmenroth

Simmenroth_A@ukw.de

Stefanie Stark: https://orcid.org/0000-0002-5349-7984

Stefanie.Stark@uk-erlangen.de

Lisette Warkentin: https://orcid.org/0000-0002-6241-6082

Lisette.Warkentin@uk-erlangen.de

Mark H. Ebell: https://orcid.org/0000-0003-3228-2877

ebell@uga.edu

Ildikó Gágyor: https://orcid.org/0000-0002-7974-7603

For peer review only

Gagyor_i@ukw.de

For peer review only

For peer review only - http://bmjopen.bmj.com/site/about/guidelines.xhtml

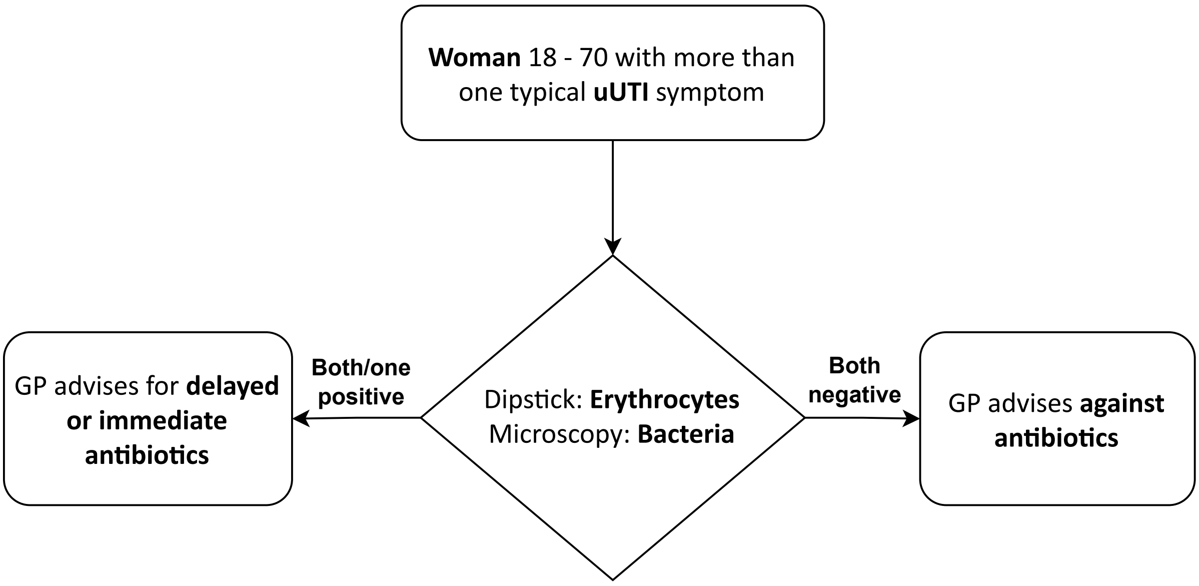
Woman 18 - 70 with more than one typical uUTI symptom
Dipstick: Erythrocytes
Microscopy: Bacteria
Both/one positive
GP advises for delayed or immediate antibiotics
Both negative
GP advises against antibiotics

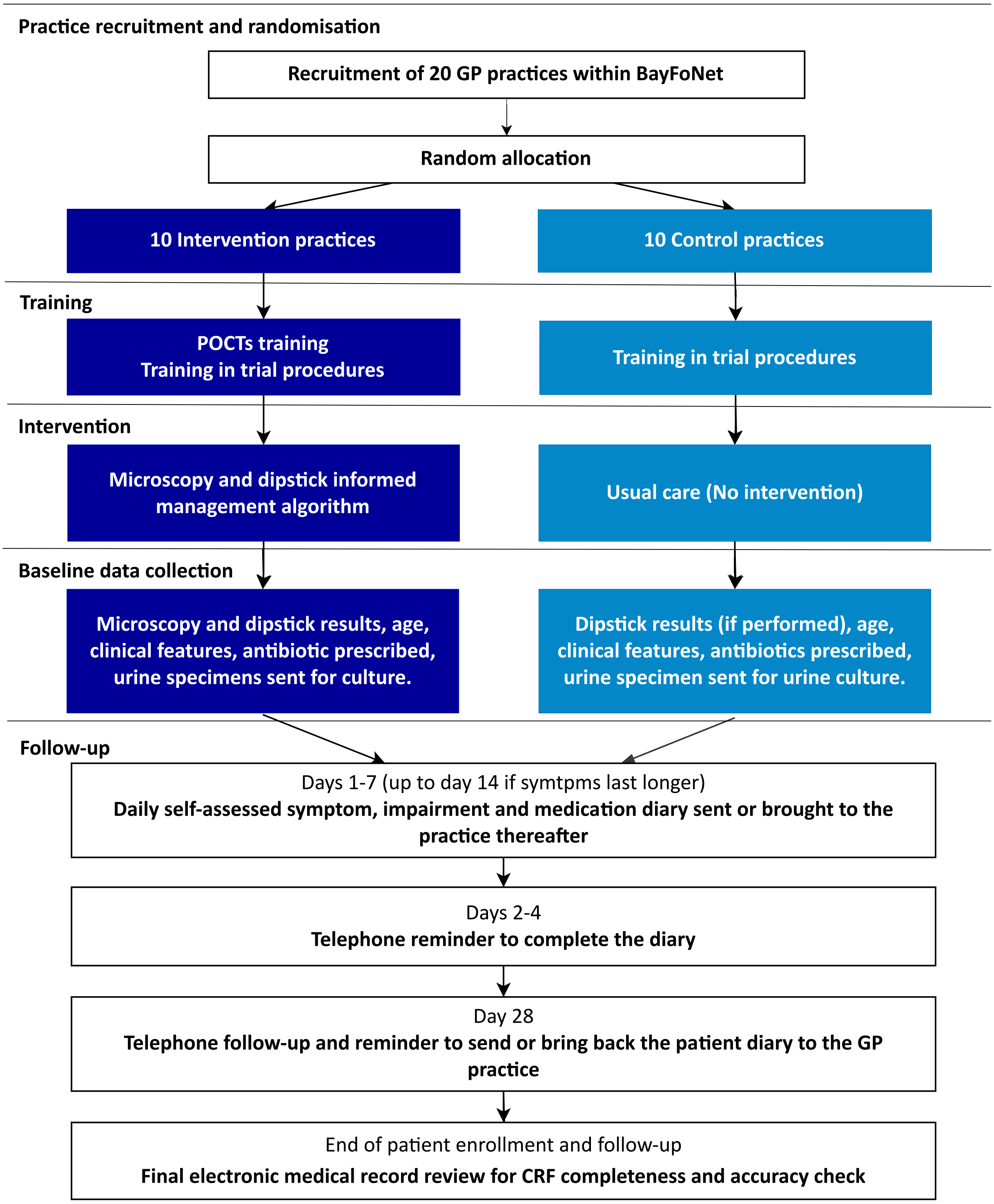
Practice recruitment and randomisation
Recruitment of 20 GP practices within BayFoNet
Random allocation
10 Intervention practices
10 Control practices
Training
POCTs training
Training in trial procedures
Training in trial procedures
Intervention
Microscopy and dipstick informed management algorithm
Usual care (No intervention)
Baseline data collection
Microscopy and dipstick results, age, clinical features, antibiotic prescribed, urine specimens sent for culture.
Dipstick results (if performed), age, clinical features, antibiotics prescribed, urine specimen sent for urine culture.
Follow-up
Days 1-7 (up to day 14 if symtpms last longer)
Daily self-assessed symptom, impairment and medication diary sent or brought to the practice thereafter
Days 2-4
Telephone reminder to complete the diary
Day 28
Telephone follow-up and reminder to send or bring back the patient diary to the GP practice
End of patient enrollment and follow-up
Final electronic medical record review for CRF completeness and accuracy check

Data management and protection

The recording, disclosure, storage and analysis of personal data within this clinical trial will take place in accordance with the legal provisions of the EU General Data Protection Regulation (GDPR) and of the Bavarian Data Protection Act ('BayDSG') of 15 May 2018. A prerequisite for this is the voluntary agreement of the participants prior to participation.
Data will be recorded and stored as follows.

Data management and protection in each study site

The recruiting academic departments will provide each study site with an investigator site file (ISF), which contains all the documents required for the clinical trial. These include (1) patient questionnaires, (2) eligibility case report forms (CRFs), (3) patient information leaflets with informed consent forms, (4) baseline CRFs, (5) patient diaries, (6) follow-up CRFs, (7) a pseudonymisation list, (8) a patient contact form, and (9) a pre-screening list.
Medical assistants in each study site will be instructed to fill in the pseudonymisation list by consecutively adding the name, birthdays, and date of inclusion of participants. Next to the name, medical assistants will be instructed to write down a patient-ID, containing the letter W or E to identify if the study centre, a three digit number to identify the study site (e.g. 001), and a three digit number to identify the patient (e.g. 001). The pseudonymisation list will be stored separately to all other trial documents and will remain at the study site until the end of data analysis, and destroyed thereafter.
Whenever a new patient is enrolled and once the consent forms are signed:

1) Documents 1, 2, 4-6, and 8 will be provided with the pseudonymized patient-ID.
2) The patient contact information (i. e. telephone number, together with the patient-ID) is written down on the patient contact form and data are entered on REDCap to be transmitted the same day to either the Institute of General Practice of the Friedrich-Alexander Universität Erlangen-Nürnberg (FAU; practices located in or near Erlangen), or to the Institute of General Practice and Family Medicine of the Ludwig-Maximilians University Hospital Munich (LMU; practices located in or near Wuerzburg). It is then stored in the Investigator site file (ISF).
3) The dip-slide is provided with the pseudonymized patient-ID and sent to the Institute of Hygiene and Microbiology of the Julius-Maximilians-University of Würzburg (IMH) via traditional post for microbiological analyses. All results will be reported individually to the treating physician, who will store the report in the ISF. A copy of every report is additionally sent to the trial coordinating centre (UKW).

ISF-data transfer to the academic departments

Researchers and research nurses of the trial teams of the UKW (when practices are located in or near Würzburg) and the FAU (when practices are located in or near Erlangen) perform outreach visits to each study site to collect the paper-based documents (except for the pseudonymisation lists) containing patient data.

Data management and protection at the Academic departments

Research nurses at the trial centres enter the following data in a browser-based electronic Research Data Capture system (REDCap), a password-protected online storage system:

- at the UKW and the FAU they enter all paper-based data of the ISF;
- at the UKW they enter the results of the microbiological analyses performed at the IHM

The Institute of Clinical Epidemiology and Biometry is responsible for providing access to and hosting the EDC-System REDCap, including user management and to ensure data security by regular backups of the database in the context of BayFoNet.

Regular plausibility, validity and consistency checks will be carried out on the described electronic CRFs. Once the data collection is completed, all entries have been made, and final plausibility checks have been carried out, the databases will be closed.

Data management and protection of patients who are not included in the trial

To ensure consecutive enrolment and control for recruitment bias, the following management is planned also for data of patients not subsequently included in the trial:

1) In each study site, all potential participants are approached and listed in an anonymous paper-based pre-screening logs.
2) All approached patients will be asked to complete a symptom questionnaire (document 1, paragraph "Data management and protection in each study site"). Questionnaires filled by included women will be provided with the participant-ID, whereas questionnaires of non-participants will not contain information which could lead to an identification (=*anonymous* data).
3) During each consultation with potentially eligible patients, the GP fills the eligibility CRF (document 2, paragraph "Data management and protection in each study site"). Only data of included women will be provided with the participant-ID. Eligibility CRFs of non-participants will not contain information which could lead to an identification (=*anonymous* data).

These documents will be handed out to the trial teams of the UKW or the FAU together with the ISFs during the final outreach visit.

Storage of paper-based documents at the trial centres

All paper-based documents will be securely stored at the UKW and the FAU and will be accessible only to authorized individuals for ten years and destroyed thereafter.

Storage of human biosamples

No biosamples will be stored in this trial. In the study sites, urine specimens are securely disposed, as in usual clinical practice, immediately after POCTs have been performed. Dip-slides provided with the pseudonymized patient-ID sent to the IMH can be used only for the microbiological analyses as outlined above, and will be disposed thereafter.

For peer review only

SPIRIT
STANDARD PROTOCOL ITEMS: RECOMMENDATIONS FOR INTERVENTIONAL TRIALS

SPIRIT 2013 Checklist: Recommended items to address in a clinical trial protocol and related documents*

Section/item	Item No	Description	Addressed on page number
**Administrative information**			
Title	1	Descriptive title identifying the study design, population, interventions, and, if applicable, trial acronym	Title page 1
Trial registration	2a	Trial identifier and registry name. If not yet registered, name of intended registry	Abstract p. 2
	2b	All items from the World Health Organization Trial Registration Data Set	Abstract p. 2
Protocol version	3	Date and version identifier	Abstract p. 2
Funding	4	Sources and types of financial, material, and other support	P. 14
Roles and responsibilities	5a	Names, affiliations, and roles of protocol contributors	Title page 1
	5b	Name and contact information for the trial sponsor	Title page 1
	5c	Role of study sponsor and funders, if any, in study design; collection, management, analysis, and interpretation of data; writing of the report; and the decision to submit the report for publication, including whether they will have ultimate authority over any of these activities	P. 14
	5d	Composition, roles, and responsibilities of the coordinating centre, steering committee, endpoint adjudication committee, data management team, and other individuals or groups overseeing the trial, if applicable (see Item 21a for data monitoring committee)	P. 13 and 14 where applicable

For peer review only

Introduction

Background and rationale	6a	Description of research question and justification for undertaking the trial, including summary of relevant studies (published and unpublished) examining benefits and harms for each intervention	P. 3 and 4
	6b	Explanation for choice of comparators	P. 9
Objectives	7	Specific objectives or hypotheses	P. 4 and 5
Trial design	8	Description of trial design including type of trial (eg, parallel group, crossover, factorial, single group), allocation ratio, and framework (eg, superiority, equivalence, noninferiority, exploratory)	P. 4

Methods: Participants, interventions, and outcomes

Study setting	9	Description of study settings (eg, community clinic, academic hospital) and list of countries where data will be collected. Reference to where list of study sites can be obtained	P. 5
Eligibility criteria	10	Inclusion and exclusion criteria for participants. If applicable, eligibility criteria for study centres and individuals who will perform the interventions (eg, surgeons, psychotherapists)	P. 6
Interventions	11a	Interventions for each group with sufficient detail to allow replication, including how and when they will be administered	P. 7 - 9
	11b	Criteria for discontinuing or modifying allocated interventions for a given trial participant (eg, drug dose change in response to harms, participant request, or improving/worsening disease)	NA
	11c	Strategies to improve adherence to intervention protocols, and any procedures for monitoring adherence (eg, drug tablet return, laboratory tests)	P. 7 (training)
	11d	Relevant concomitant care and interventions that are permitted or prohibited during the trial	NA
Outcomes	12	Primary, secondary, and other outcomes, including the specific measurement variable (eg, systolic blood pressure), analysis metric (eg, change from baseline, final value, time to event), method of aggregation (eg, median, proportion), and time point for each outcome. Explanation of the clinical relevance of chosen efficacy and harm outcomes is strongly recommended	P. 4 and 5
Participant timeline	13	Time schedule of enrolment, interventions (including any run-ins and washouts), assessments, and visits for participants. A schematic diagram is highly recommended (see Figure)	P. 10, Table 1 and P. 11 Figure 2

For peer review only - http://bmjopen.bmj.com/site/about/guidelines.xhtml

Sample size	14	Estimated number of participants needed to achieve study objectives and how it was determined, including clinical and statistical assumptions supporting any sample size calculations	P. 12 and 13
Recruitment	15	Strategies for achieving adequate participant enrolment to reach target sample size	P. 7 (training)
**Methods: Assignment of interventions (for controlled trials)**			
Allocation:			
Sequence generation	16a	Method of generating the allocation sequence (eg, computer-generated random numbers), and list of any factors for stratification. To reduce predictability of a random sequence, details of any planned restriction (eg, blocking) should be provided in a separate document that is unavailable to those who enrol participants or assign interventions	P. 6
Allocation concealment mechanism	16b	Mechanism of implementing the allocation sequence (eg, central telephone; sequentially numbered, opaque, sealed envelopes), describing any steps to conceal the sequence until interventions are assigned	NA
Implementation	16c	Who will generate the allocation sequence, who will enrol participants, and who will assign participants to interventions	P. 6
Blinding (masking)	17a	Who will be blinded after assignment to interventions (eg, trial participants, care providers, outcome assessors, data analysts), and how	P. 6 (analyst blinding)
	17b	If blinded, circumstances under which unblinding is permissible, and procedure for revealing a participant's allocated intervention during the trial	NA
**Methods: Data collection, management, and analysis**			
Data collection methods	18a	Plans for assessment and collection of outcome, baseline, and other trial data, including any related processes to promote data quality (eg, duplicate measurements, training of assessors) and a description of study instruments (eg, questionnaires, laboratory tests) along with their reliability and validity, if known. Reference to where data collection forms can be found, if not in the protocol	P 9 and 10
	18b	Plans to promote participant retention and complete follow-up, including list of any outcome data to be collected for participants who discontinue or deviate from intervention protocols	P. 9

Data management	19	Plans for data entry, coding, security, and storage, including any related processes to promote data quality (eg, double data entry; range checks for data values). Reference to where details of data management procedures can be found, if not in the protocol	P.12, 13
Statistical methods	20a	Statistical methods for analysing primary and secondary outcomes. Reference to where other details of the statistical analysis plan can be found, if not in the protocol	P. 12
	20b	Methods for any additional analyses (eg, subgroup and adjusted analyses)	NA (Line 409-410)
	20c	Definition of analysis population relating to protocol non-adherence (eg, as randomised analysis), and any statistical methods to handle missing data (eg, multiple imputation)	P. 12
**Methods: Monitoring**			
Data monitoring	21a	Composition of data monitoring committee (DMC); summary of its role and reporting structure; statement of whether it is independent from the sponsor and competing interests; and reference to where further details about its charter can be found, if not in the protocol. Alternatively, an explanation of why a DMC is not needed	NA (complex intervention, not drug trial)
	21b	Description of any interim analyses and stopping guidelines, including who will have access to these interim results and make the final decision to terminate the trial	NA
Harms	22	Plans for collecting, assessing, reporting, and managing solicited and spontaneously reported adverse events and other unintended effects of trial interventions or trial conduct	NA
Auditing	23	Frequency and procedures for auditing trial conduct, if any, and whether the process will be independent from investigators and the sponsor	NA
**Ethics and dissemination**			
Research ethics approval	24	Plans for seeking research ethics committee/institutional review board (REC/IRB) approval	P. 13
Protocol amendments	25	Plans for communicating important protocol modifications (eg, changes to eligibility criteria, outcomes, analyses) to relevant parties (eg, investigators, REC/IRBs, trial participants, trial registries, journals, regulators)	P. 13

For peer review only - http://bmjopen.bmj.com/site/about/guidelines.xhtml

Consent or assent	26a	Who will obtain informed consent or assent from potential trial participants or authorised surrogates, and how (see Item 32)	P. 6 and 7
	26b	Additional consent provisions for collection and use of participant data and biological specimens in ancillary studies, if applicable	NA
Confidentiality	27	How personal information about potential and enrolled participants will be collected, shared, and maintained in order to protect confidentiality before, during, and after the trial	P. 13
Declaration of interests	28	Financial and other competing interests for principal investigators for the overall trial and each study site	P. 14
Access to data	29	Statement of who will have access to the final trial dataset, and disclosure of contractual agreements that limit such access for investigators	P. 14
Ancillary and post-trial care	30	Provisions, if any, for ancillary and post-trial care, and for compensation to those who suffer harm from trial participation	NA
Dissemination policy	31a	Plans for investigators and sponsor to communicate trial results to participants, healthcare professionals, the public, and other relevant groups (eg, via publication, reporting in results databases, or other data sharing arrangements), including any publication restrictions	P 13
	31b	Authorship eligibility guidelines and any intended use of professional writers	NA
	31c	Plans, if any, for granting public access to the full protocol, participant-level dataset, and statistical code	P 14
**Appendices**			
Informed consent materials	32	Model consent form and other related documentation given to participants and authorised surrogates	Not intended for publication
Biological specimens	33	Plans for collection, laboratory evaluation, and storage of biological specimens for genetic or molecular analysis in the current trial and for future use in ancillary studies, if applicable	P 11-12

*It is strongly recommended that this checklist be read in conjunction with the SPIRIT 2013 Explanation & Elaboration for important clarification on the items. Amendments to the protocol should be tracked and dated. The SPIRIT checklist is copyrighted by the SPIRIT Group under the Creative Commons "Attribution-NonCommercial-NoDerivs 3.0 Unported" license.
